# Increased Autoreactive CD8^+^ T Cells to Apolipoprotein B Epitopes in Patients With Severe Coronary Artery Disease

**DOI:** 10.1161/CIRCRESAHA.125.326804

**Published:** 2026-07-23

**Authors:** Payel Roy, Smriti Parashar, John Sidney, Megh Mehta, Ishita Tandon, Margaret Beddall, Yan Wang, Irina Baranovskaya, Andrew Soo Hoo, Ralph Ierardi, Gautam Agarwal, William D. Jordan, Coleen A. McNamara, Alessandro Sette, Hossam Abdelsamed, Klaus Ley

**Affiliations:** 1 Immunology Center of Georgia, Augusta University 1421 , Augusta (P.R., S.P., M.M., I.T., M.B., Y.W., I.B., H.A., K.L.).; 2 Center for Inflammation Biology (P.R., K.L.), La Jolla Institute for Immunology, CA.; 3 Center for Infectious Disease and Vaccine Research (J.S., A.S.), La Jolla Institute for Immunology, CA.; 4 Now with Department of Biochemistry, Indian Institute of Science, Bengaluru, India (P.R.).; 5 Department of Surgery (A.S.H., R.I., G.A., W.D.J.), Medical College of Georgia at Augusta University.; 6 Department of Physiology (H.A., K.L.), Medical College of Georgia at Augusta University.; 7 Department of Medicine/Cardiovascular Division and Carter Immunology Center, University of Virginia 2358 , Charlottesville (C.A.M.N.).; 8 Department of Medicine (A.S.), University of California, San Diego, La Jolla.; 9 Department of Bioengineering (K.L.), University of California, San Diego, La Jolla.

**Keywords:** atherosclerosis, humans, immunodominant epitopes, major histocompatibility complex, staining and labeling

## Abstract

**BACKGROUND:**

Atherosclerosis is a chronic inflammatory disease with a strong autoimmune component, marked by the detection of autoreactive T cells and autoantibodies. Recent single-cell RNA sequencing studies have shown that atherosclerotic plaques contain clonally expanded CD8^+^ T cells. One of the known atherosclerosis autoantigens is APOB (apolipoprotein B). However, autoreactive CD8^+^ T cells to APOB in humans have not been described.

**METHODS:**

We studied CD8^+^ T-cell reactivity to human leukocyte antigen-A*02:01-restricted APOB epitopes, starting with in silico epitope prediction. We used peripheral blood mononuclear cells from human leukocyte antigen-A02:01+ healthy subjects to test the top 64-ranked peptides for their potential to elicit a CD8^+^ T-cell response. Antigen-specific responses were assessed using activation-induced marker assays, intracellular cytokine staining, and IFNγ (interferon gamma) ELISpot assays.

**RESULTS:**

Some APOB peptides triggered robust CD8^+^ T-cell activation with effector memory features, and expression of cytokines and cytotoxic molecules. Five immunodominant epitopes spanning 2 APOB regions accounted for most of the response and elicited significant T-cell activation in healthy donors that was increased in clinical samples from patients with severe coronary artery disease.

**CONCLUSIONS:**

The discovery of immunodominant major histocompatibility complex class I-restricted APOB epitopes suggests a new perspective for immune-based interventions to mitigate atherosclerosis.

**Novelty and Significance**

**What Is Known?**

- CD8^+^ T cells are the most abundant T cells in human atherosclerotic plaque and have the potential to influence vascular inflammation; however, the epitopes they recognize remain largely unknown.
- ApoB (apolipoprotein B), the major protein component of atherogenic lipoproteins, can act as an autoantigen in human atherosclerosis.
- Identifying the peptide epitopes recognized by autoreactive CD8^+^ T cells is essential for understanding their role in disease.

**What New Information Does This Article Contribute?**

- Systematic screening of ≈4600 ApoB peptides predicted to bind the common major histocompatibility complex class I allele human leukocyte antigen-A*02:01 identified 5 immunodominant ApoB epitopes.
- These 5 immunodominant ApoB peptides contain 2 core peptide sequences (SLLPQLIEV and TLLDSPIKV), revealing that human CD8^+^ T-cell recognition of ApoB is focused on a limited set of antigenic determinants.
- Together, these epitopes account for approximately half of detectable ApoB-specific CD8^+^ T-cell responses in human peripheral blood.
- Patients with severe coronary artery disease showed stronger ApoB-specific expansion of their CD8^+^ T cells than patients with no or mild coronary artery disease.

We identified the presence of APOB-specific human leukocyte antigen-A*02:01-restricted autoreactive CD8^+^ T cells in human peripheral blood. These cells were detectable in healthy individuals and were increased in patients with severe coronary artery disease. A systematic screening and deconvolution approach led to the identification of 5 immunodominant APOB epitopes that accounted for most of these responses. These findings provide a basis for developing antigen-specific approaches to interrogate autoreactive CD8^+^ T-cell responses in human atherosclerosis.

**Meet the First Author, see p e000767**

How the immune system recognizes antigens has been an intriguing question for a long period of time. The early discovery work by Zinkernagel and Doherty established the concept of major histocompatibility complex class I (MHC-I) restriction, where CD8^+^ T cells primarily recognize endogenous peptides bound to MHC-I.^1^ Consequently, microbial and tumor peptides can be targeted by cytotoxic CD8^+^ T cells in an MHC-I-dependent manner. Self-peptides can trigger an autoimmune response, giving rise to a wide spectrum of autoimmune diseases.

Recent studies have shown that atherosclerosis is associated with loss of tolerance and aberrant activation of autoreactive T cells,^2–5^ highlighting the involvement of vigorous T-cell responses to epitopes from endogenous proteins. Tetramer-identified CD4^+^ T cells specific for self-peptides in naïve mice express PD-1 and CD73, which limits the response to self.^6^ Although CD8^+^ T cells are best known for providing immune protection, their inappropriate activation can have severe consequences, as reported in the pathogenesis of autoimmune diseases such as type 1 diabetes, rheumatoid arthritis, and multiple sclerosis.^7–9^ Despite the plethora of autoimmune diseases, the nature of the autoantigen(s) in many of them is still unknown. In atherosclerosis, a chronic inflammatory vascular disease characterized by immune cell infiltration and plaque formation,^2,10,11^ CD8^+^ T cells are found in blood and plaques, but the specificity of atherosclerosis-related autoreactive CD8^+^ T cells is unknown.

Here, we used a recently established peptide stimulation-based assay to detect human circulating autoreactive T cells.^12,13^ The adaptive immune response to ApoB-100 (apolipoprotein B), the core protein in atherogenic molecules like low-density lipoprotein, is a hallmark of atherosclerosis,^14^ which makes ApoB-100 peptides potential targets for CD8^+^ T cells. Here, we first took an in silico approach to predict the potential human leukocyte antigen (HLA)-A*02:01-restricted epitopes spanning the human APOB protein. We then distributed the identified high-affinity peptides into mesopools. Peripheral blood mononuclear cells (PBMCs) were obtained from HLA-A*02:01^+^ donors, and the stimulation capacity of each mesopool was examined by measuring the upregulation of activation-induced markers (AIMs), including CD25, CD69, and 4-1BB, on CD8^+^ T cells using flow cytometry. Intracellular cytokine staining (ICS) assays detected APOB peptide-induced expression of inflammatory and cytotoxic molecules in human CD8^+^ T cells. The antigenicity of individual epitopes was screened in an IFNγ (interferon gamma) ELISpot assay. The CD8^+^ T-cell response to the 5 immunodominant peptides was confirmed in additional HLA-A*02:01^+^ healthy controls and HLA-A*02:01^+^ patients with coronary artery disease (CAD) or those undergoing surgical endarterectomy using the validated AIM assay.

## Methods

### Data Availability

All data, details of methods and essential research materials used for the research and analysis have been provided in this article. The data that support the findings of this study are available from the corresponding authors upon reasonable request.

### Human Subjects

Healthy participants, enrolled in the Normal Blood Donation Program, were recruited through the clinical core at the La Jolla Institute for Immunology. Written informed consent was obtained from all participants. Donors were negative for any significant systemic disease and viral infections, including hepatitis B or C and HIV. Ethical approval for the study was provided by the La Jolla Institute for Immunology’s institutional review board (protocol no. VD-057, IB-248-0821). Donor HLA information was obtained as previously described.^12^ Briefly, the REPLI-g DNA Midi Kit (QIAGEN, Hilden, Germany) was used to isolate genomic DNA from human PBMCs using manufacturer’s instructions. DNA samples were sent to an American Society for Histocompatibility and Immunogenetics-accredited laboratory at the Institute for Immunology & Infectious Diseases, Murdoch University, Western Australia. Mapped HLA allele information was obtained.

For the clinical Coronary Assessment in Virginia cohort, blood samples were collected from 8 individuals undergoing cardiac catheterization at the University of Virginia Health System in Charlottesville, Virginia (Table S3). All participants provided written informed consent before enrollment, and blood specimens were obtained before the catheterization procedure. The study protocol was reviewed and approved by the University of Virginia Human institutional review board (No. 15328).

Quantitative coronary angiography was performed using automatic edge detection from an end-diastolic frame, which was selected for each lesion based on the demonstration of the most severe stenosis with minimal foreshortening and branch overlap. The minimum lumen diameter, reference diameter, percent diameter stenosis, and stenosis length were calculated by blinded, experienced investigators, who assessed disease severity based on the Gensini score. Each coronary lesion received a severity score ranging from 0 to 32 according to the degree of luminal narrowing. This value was subsequently weighted by a factor between 0.5 and 5 based on the anatomic location of the lesion. Weighted scores from all coronary segments were summed to generate the overall angiographic CAD disease score. Score adjustment for collateral circulation was not performed for this study. Mapped HLA allele information for these patients was obtained as described for the healthy donors.

For the Augusta University clinical cohort, 6 patients undergoing endarterectomy at the Medical College of Georgia Department of Surgery were enrolled (clinical and demographic details are shown in Table S4). On the day of surgery, blood samples were collected before the procedure. Written informed consent was obtained from all participants. Ethical approval for the study was provided by Augusta University (IRBNet ID: 2088330-10). Single locus HLA-A typing for the Augusta University cohort was done by CD Genomics, New York.

For both healthy and clinical cohorts, PBMCs were isolated from the freshly collected blood specimens as described later and cryopreserved in the freezing medium containing DMSO at −196 °C on liquid nitrogen until further use. PBMCs from the Coronary Assessment in Virginia cohort were transported to Augusta University by overnight shipment on dry ice.

### In Silico Prediction of HLA*02:01 Binding Epitopes

We used prediction tools available from the Immune Epitope Database.^15^ Potential epitopes, spanning the entire length of human APOB (4563 amino acids), were scored for binding to HLA-A*02:01 using NetMHCpanEL-4.0.^16^ This algorithm is trained on data from both binding affinity and eluted ligands and adjusts for peptide length preferences. Among the scored peptides, 9-mers and 10-mers were most common, but longer peptides were also found. The tool transforms predicted peptide binding affinities (50% inhibitory concentrations, IC50 values) into Immune Epitope Database percentile scores. As high-affinity ligands typically lie within the top 0.5%, we ranked the epitopes according to their scores and selected the top 64, which lay within the top 0.17% of the ranked APOB peptides. This epitope set contained forty-one 9-mers, eighteen 10-mers, two 12-mers, two 13-mers and one 14-mer.

### Peptides

The selected 64 peptides (details are provided in Table S1) were synthesized at >70% purity by TC Peptide Lab (San Diego). Peptides within each mesopool were resuspended in dimethyl sulfoxide (DMSO, Sigma-Aldrich) and combined in equal concentrations to prepare the APOB Class I mesopools A (peptides 1–16), B (peptides 17–32), C (peptides 33–48), and D (peptides 49–64). After epitope discovery, 5 immunodominant APOB Class I peptides (referred to as APOB_5_) were pooled for confirmatory experiments in patients with CAD, endarterectomy subjects and healthy controls (final concentration of each individual peptide, 10 µg/mL).

### PBMC Isolation by Density-Gradient Centrifugation

Venous blood samples were collected in tubes coated with K2-EDTA anticoagulant. The tubes were centrifuged at 800×*g* for 15 minutes at room temperature with the brake off. The top layer of plasma was removed, and an equivalent amount of 1× phosphate-buffered saline (PBS, w/o Ca/Mg, Gibco) was added, and the sample was resuspended thoroughly. Seven parts of diluted blood were gently layered on 3 parts of Ficoll-Paque Plus (Millipore Sigma) and centrifuged at 800×*g* for 30 minutes at room temperature with the brake off. The layer of cells at the interface was carefully harvested and washed twice with 1× PBS. Cell count and viability were evaluated using the Trypan Blue dye exclusion method. PBMCs were cryopreserved in freezing medium with DMSO at −196 °C on liquid nitrogen until further use.

### Stimulation Assays and Flow Cytometry

Human PBMCs were cultured in serum-free cell culture medium (TexMACS, Miltenyi Biotec) supplemented with 1% penicillin/streptomycin (Thermo Fisher Scientific). Cells were plated at a density of 2×10^6^ cells/mL in 24-well plates and were stimulated with each mesopool at a concentration of 5 to 10 μg/mL per peptide. For validation experiments, PBMCs from healthy or clinical subjects were stimulated with a pool of 5 identified immunodominant APOB epitopes (APOB_5_; final concentration of each individual peptide in the pool, 10 µg/mL). For specificity experiments, cells were expanded with CEFX-I (Ultra SuperStim Pool, MHC-I, JPT Peptide Technologies; a pool of 80 peptides; final concentration of each individual peptide in the pool, 1.5 µg/mL). Subsequently, cells were incubated at 37 °C with 5% CO_2_. Human IL-2 (10 U/mL; Invitrogen) was added on days 4, 7, and 10. After 14 days, PBMCs were harvested and washed with PBS. Cells were replated in 96-well plates at 1.5×10^6^ cells per well and restimulated with the cognate mesopool (5–10 μg/mL, each peptide) or APOB_5_ peptide pool (10 μg/mL, each peptide), or CEFX-I (1.5 μg/mL, each peptide). Unstimulated sets served as controls for background subtraction of peptide-induced responses. To conduct the AIM assay, cells were treated with 1 μg/mL of a CD40-blocking antibody (Novus Biologicals) 15 minutes before mesopool stimulation. Cells were incubated for 24 hours at 37 °C with 5% CO_2_. In the ICS assay, 1× protein transport inhibitor cocktail (eBioscience) was added 2 hours after mesopool stimulation and incubated for an additional 4 hours at 37 °C with 5% CO_2_. At the end of the stimulation phase (24 hours for the AIM assay or 6 hours for the ICS assay), cells were washed with cold flow cytometry buffer (PBS w/o Ca^2+^/Mg^2+^, 2% fetal bovine serum [FBS]) and resuspended in a staining master mix containing human Fc-Block, fixable viability dye and fluorochrome-conjugated antibodies to identify T cells (CD3, CD4, CD8) and to exclude non-T cells (CD14, CD16, CD19, CD56).

Additionally, fluorochrome-conjugated antibodies against T-cell activation markers (CD40L, CD25, CD69, 4-1BB, OX-40) and naïve/memory markers (CCR7, CD45RA) were added to identify naïve, central memory T-cell and effector memory T-cell subsets. Cells were incubated with antibodies against surface markers for 45 minutes at 4 °C and then washed with cold flow cytometry buffer (PBS w/o Ca/Mg, 2% FBS). For intracellular cytokine detection (only in the ICS assay), cells were fixed with IC Fixation Buffer (eBioscience) for 30 minutes at 24 °C. Fixed cells were washed, permeabilized, and stained in 1× Perm Buffer solution (eBioscience) with fluorochrome-conjugated antibodies to cytokines (TNF [tumor necrosis factor], IFNγ) and cytotoxic markers (perforin, granzyme B) for 45 minutes at 24 °C. Cells were washed with 1× Perm Buffer and analyzed on an LSR II (BD Biosciences) flow cytometer. Single-color stained beads (UltraComp eBeads, Invitrogen) were used for compensation. Data were analyzed with FlowJo (v10.8.1). For the experiments performed with the APOB_5_ pool, data were collected on a Cytek Aurora 5-Laser Spectral Cytometer and analyzed with FlowJo (version 10.10.0).

### Enzyme-Linked Immunospot (ELISpot) Assay

Human PBMCs were cultured in the presence of APOB mesopools using the protocol described above. After 14 days, cells were harvested, washed, and plated in 96-well PVDF-ELISpot plates (Millipore) coated with 5 µg/mL mouse anti-human IFNγ (clone 1-D1K) antibody (Mabtech).

PBMCs were stimulated with the cognate mesopool (10 µg/mL) or with individual peptides (20 µg/mL) in that mesopool. Unstimulated and phytohemagglutinin-L (eBioscience)-stimulated cells were used as negative and positive controls, respectively. After 24 hours of incubation at 37 °C, plates were washed 6 times with PBS containing 0.05% Tween 20 (Millipore Sigma). Plates were then incubated with 1µg/mL mouse anti-human IFNγ (clone 7-B6-1) biotinylated antibody (Mabtech) in PBS containing 0.5% BSA (Millipore Sigma) for 2 hours at room temperature. Plates were again washed 6× with PBS/0.05% Tween 20. and incubated with VECTASTAIN Elite ABC-HRP Kit, Peroxidase (Vector Laboratories) in PBS/0.1% Tween 20 for 1 hour at room temperature. Plates were washed 6× with ultrapure distilled water (Invitrogen). Plates were then incubated with 3-amino-9-ethylcarbazole (Millipore Sigma) tablets dissolved in *N*,*N*-dimethylformamide (Millipore Sigma), acetate buffer, and hydrogen peroxide solution (0.015%, Millipore Sigma) for 10 minutes at room temperature to develop secreted cytokine spots (brown). Finally, plates were dried, wells were imaged, and spot-forming cells (SFCs) were quantified using the AID (Autoimmun Diagnostika) ELISpot reader.

In experiments involving HLA blockade, HLA-DR (clone L243), DP (clone B7/21), DQ (clone SVPL3), or pan-HLA class I (W6/32) monoclonal antibodies (kindly provided by Dr Alessandro Sette, La Jolla Institute for Immunology) were added at a concentration of 10 µg/mL, 30 minutes before restimulation with peptides.

### Statistical Analyses

Data analysis and statistical comparisons were done using GraphPad Prism, v10.0.0. Two-sample comparisons were done with the nonparametric 2-tailed Wilcoxon matched-pairs signed-rank test (for paired comparisons) or the Mann-Whitney *U* test (for unpaired comparisons). We used the Kruskal-Wallis test with Dunn multiple-comparisons testing for analyses involving >2 samples. A 2-way ANOVA with the Dunnett multiple-comparisons test was performed when 2 independent variables were involved. All statistical tests, sample sizes, *P* values, and error bar descriptions are detailed in the legends to the respective figures.

## Results

### HLA-A*02:01 Binding APOB Peptides Activate Autoreactive CD8^+^ T Cells in Healthy Human PBMCs

Studying antigen-specific human CD8^+^ T cells requires consideration of specific HLA-I alleles because: (1) the binding of epitopes to HLA-I molecules is essential for CD8^+^ T-cell activation^17^ and (2) HLA-I molecules bind a largely nonoverlapping epitope repertoire due to their closed peptide-binding pockets.^18^ We focused on the HLA-A*02:01 allele, the most commonly occurring class I allele in North America.^19^ To gain a deeper insight into the nature of HLA-A*02:01-restricted human APOB peptides, we scored all potential epitopes, spanning the entire length of human APOB (4563 amino acids), for binding to HLA-A*02:01 using the prediction tool, NetMHCpanEL-4.0,^16^ from the Immune Epitope Database.^15^ Typically, high-affinity ligands lie within the top 0.5%.^20^ We selected the highest-scoring 64 epitopes, all of which lay within the top 0.17% of ranked APOB peptides (Table S1).

**Figure 1.**
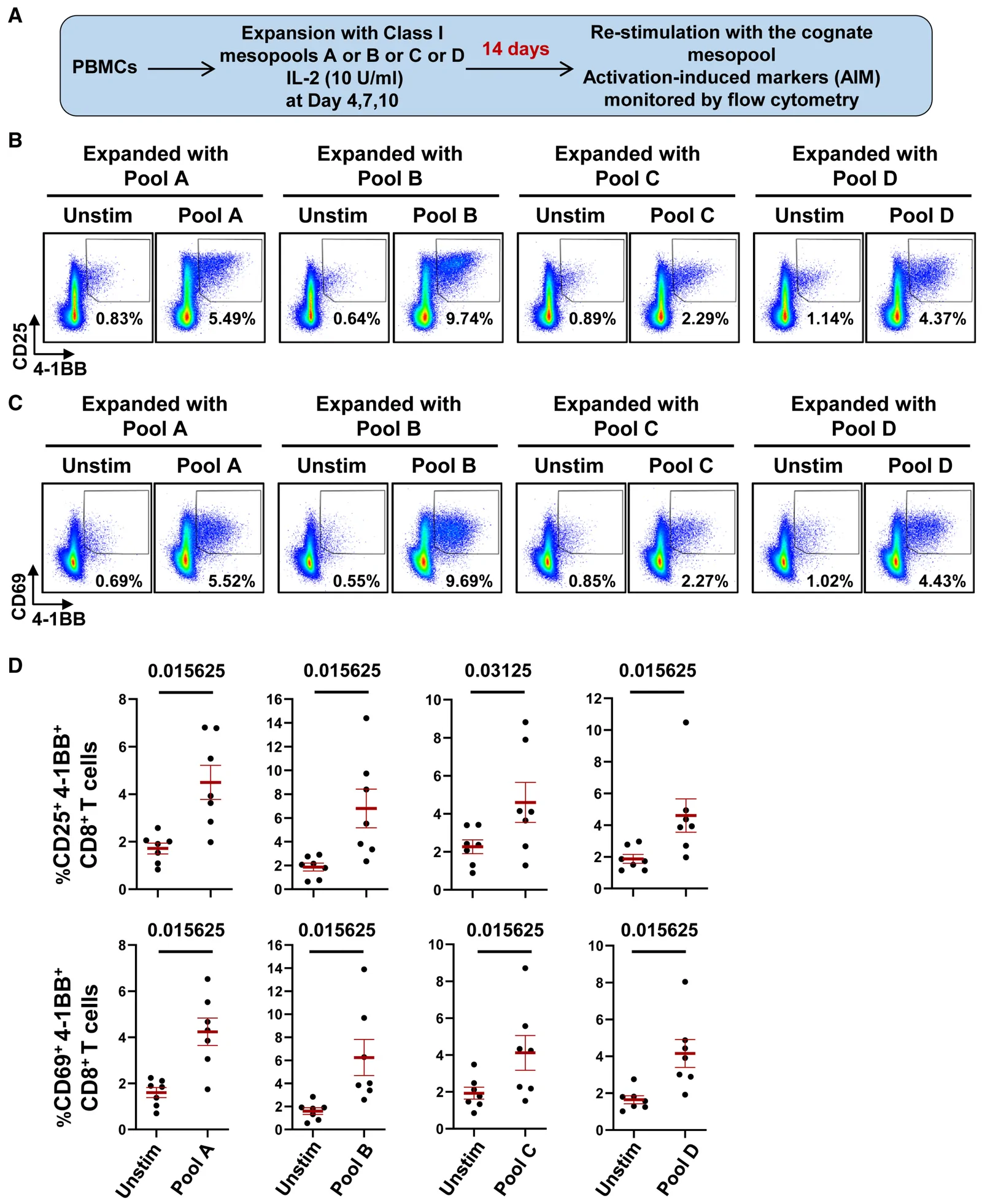
**Activation-induced marker (AIM) assay to detect APOB (apolipoprotein B)-reactive CD8^+^ T cells in healthy human peripheral blood mononuclear cells (PBMCs). A**, Schematic diagram depicting the workflow for expansion and subsequent restimulation of human PBMCs with human APOB-derived human leukocyte antigen (HLA)-A*02:01-restricted peptide mesopools (Table S1, **A**, P1–P16; **B**, P17–P32; **C**, P33–P48; **D**, P49–P64). Antigen-dependent expression of T-cell activation markers was examined using flow cytometry. **B** and **C**, Representative flow cytometry plots showing %CD25^+^4-1BB^+^ (**B**) and %CD69^+^4-1BB^+^ (**C**) populations among CD8^+^ T cells in unstimulated and individual APOB mesopool-stimulated PBMCs. **D**, Frequency of %CD25^+^4-1BB^+^ (**top**) and %CD69^+^4-1BB^+^ (**bottom**) CD8^+^ T cells in unstimulated and individual mesopool-stimulated PBMCs. N=7 independent donors. Paired statistical comparisons (**D**) between unstimulated and stimulated groups were performed using the Wilcoxon matched-pairs signed-rank test. Numerical *P* values are listed at the top of each graph. Data are represented as mean±SEM.

Next, we synthesized these 64 peptides and distributed them into 4 mesopools (A–D), each containing 16 peptides that were arranged according to their start sites in the protein sequence (Table S1). We used these mesopools to screen for APOB-reactive CD8^+^ T-cell responses in human PBMCs using a previously optimized expansion-based workflow^12^ that improves the detection limit for rare autoreactive T cells. Briefly, HLA-A*02:01+ PBMCs from 7 healthy donors were expanded and restimulated with each mesopool, and CD8^+^ T-cell responses were examined in an AIM assay using flow cytometry (schematic in Figure 1A, gating strategy in Figure S1A). Based on previous reports,^21,22^ we assessed mesopool-induced expression of 4-1BB, in combination with CD25 or CD69 as AIM markers, on CD8^+^ T cells. We observed strong APOB peptide-induced CD8^+^ T-cell responses over unstimulated controls using CD25 and 4-1BB (Figure 1B) or CD69 and 4-1BB (Figure 1C) markers. Both AIM combinations showed significantly higher responses in APOB mesopool-stimulated CD8^+^ T cells compared with unstimulated controls (Figure 1D). CD40L and CD69 were also tested as AIM markers but yielded weaker responses (Figure S1B). Thus, expression of 4-1BB, CD25, and CD69 robustly reported APOB-induced CD8^+^ T-cell responses. OX-40, another commonly used AIM marker, was not detected on CD8^+^ T cells in our assay (Figure S1C). These findings provide evidence that APOB-reactive CD8^+^ T cells are present in the peripheral blood of healthy subjects, suggesting that they escape thymic negative selection, as previously reported for CD4^+^ T cells^12,23–25^ and B cells.^26–28^

### Most AIM^+^ APOB-Reactive CD8^+^ T Cells Express T-Cell Effector Memory Markers

**Figure 2.**
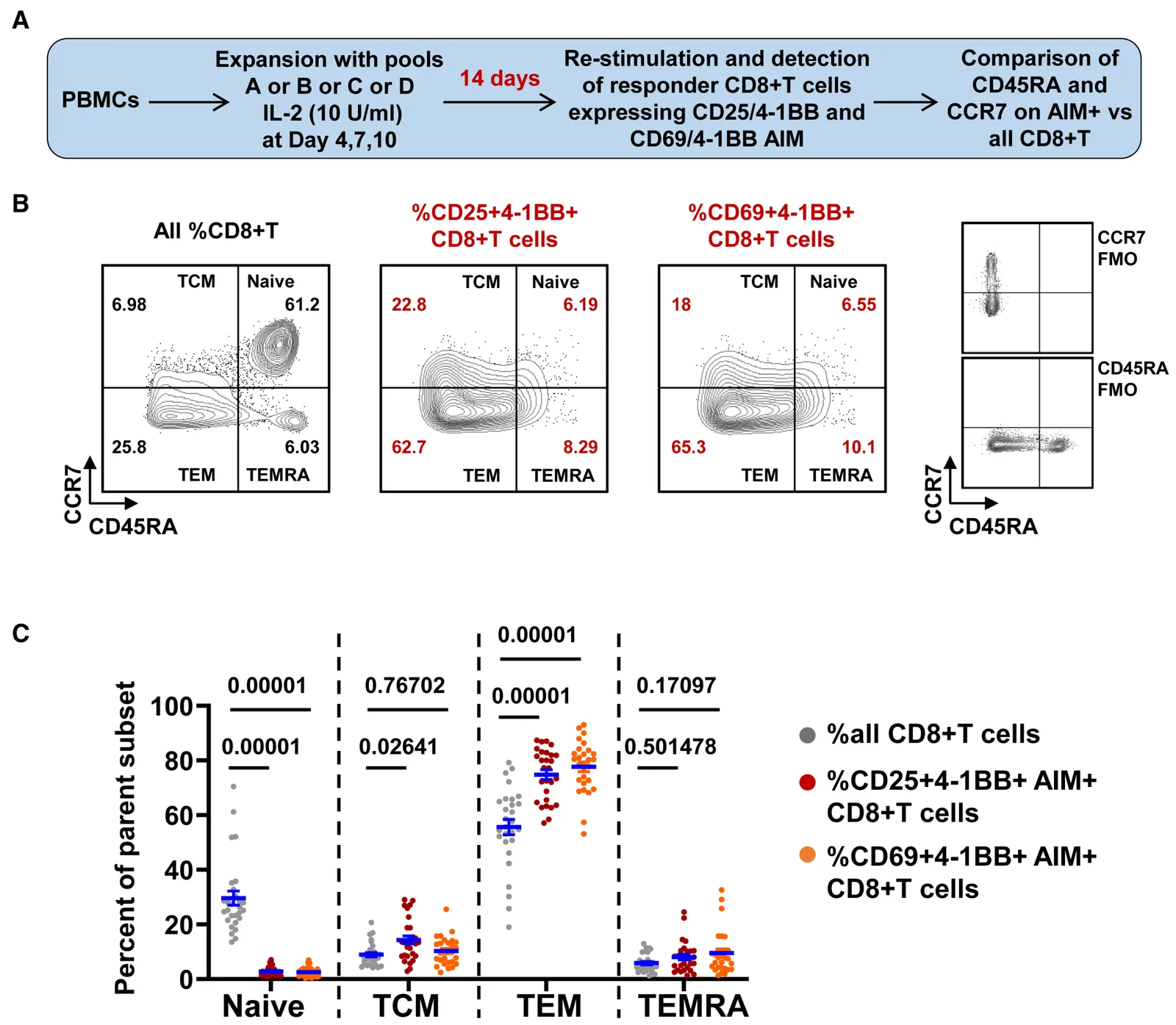
**Human APOB (apolipoprotein B)-reactive CD8^+^ T cells are enriched within the effector memory T-cell compartment. A**, Schematic representation of the workflow to examine the frequency of naïve and memory T cells within APOB-reactive CD8^+^ T cells in human peripheral blood mononuclear cells (PBMCs). **B**, Representative flow cytometry plots showing CD45RA^+^CCR7^+^ naïve, CD45RA^−^CCR7^+^ central memory (TCM), CD45RA^−^CCR7^−^ effector memory (TEM), and CD45RA^+^CCR7^−^ effector memory (TEMRA) populations among all (**left**), CD25^+^4-1BB^+^ (**middle**), and CD69^+^4-1BB^+^ (**right**) CD8^+^ T cells in PBMCs treated with APOB class I epitopes. Gates were set using fluorescence-minus-one (FMO) controls for CCR7 and CD45RA proteins. **C**, Frequency of naïve, TCM, TEM, and TEMRA subsets within all (gray), CD25^+^4-1BB^+^ (red), and CD69^+^4-1BB^+^ (orange) CD8^+^ T cells in APOB-stimulated PBMCs. Combined data from 7 independent donors, with 4 independent mesopool-stimulated sets per donor are shown. Statistical comparisons were performed using ordinary 2-way ANOVA with Dunnett multiple-comparison testing. Numerical *P* values are listed at the top of each graph. Data are represented as mean±SEM.

Having identified activated CD8^+^ T cells responding to various APOB peptides, we next sought to examine the frequency of these cells among classical naïve and memory CD8^+^ T-cell subsets. Hence, we used a combination of cell surface markers, including CCR7 and CD45RA to distinguish between the following subsets: naïve (CD45RA^+^CCR7^+^), central memory T cells (CD45RA^−^CCR7^+^), effector memory T cells (CD45RA^−^CCR7^−^), and terminally differentiated effector memory cells re-expressing CD45RA (CD45RA^+^CCR7^−^). We analyzed these markers on the APOB-reactive AIM^+^ CD8^+^ T cells and compared them with those expressed on total CD8^+^ T cells (schematic in Figure 2A). Fluorescence-minus-one controls were used to set the gates for the identification of these populations (Figure 2B, right). We observed significant enrichment of effector memory T cells within APOB-reactive CD25^+^4-1BB^+^ or CD69^+^4-1BB^+^ AIM^+^ CD8^+^ T cells as compared with bulk CD8^+^ T cells (Figure 2B, left, and Figure 2C). Few AIM^+^CD8^+^ T cells expressed naïve T-cell markers (Figure 2C). This bias toward memory phenotypes in APOB-reactive AIM+ CD8^+^ T cells was not an artifact of the assay, as both naïve and memory CD8^+^ T cells upregulated expression of these activation markers when PBMCs were stimulated with the strong T cell activator, phytohemagglutinin-L (Figure S2A and S2B). In summary, these phenotypic analyses resembled cytotoxic and inflammatory effector memory CD8^+^ T cells reported in blood and plaque lesions from patients with atherosclerotic diseases.^29–31^

### APOB-Reactive CD8^+^ T Cells Express Proinflammatory Cytokines and Cytotoxic Molecules

**Figure 3.**
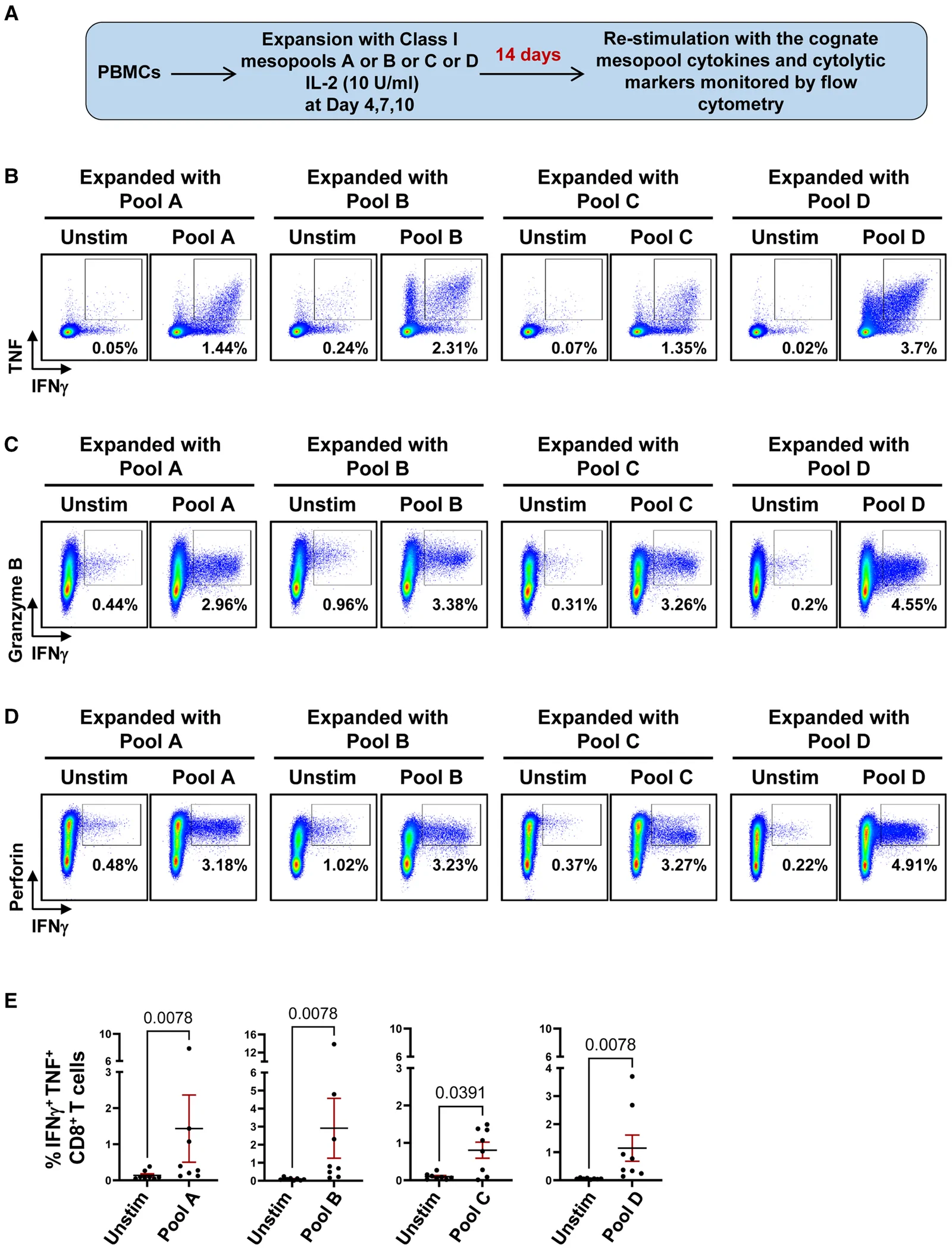
**Expression of inflammatory and cytotoxic molecules in human APOB (apolipoprotein B)-reactive CD8^+^ T cells. A**, Diagrammatic scheme showing the workflow for detection of cytokines and cytotoxic molecules using the flow cytometry-based intracellular cytokine staining (ICS) assay. **B** through **D**, Representative flow cytometry plots showing % tumor necrosis factor (TNF)^+^ interferon-gamma (IFNγ)^+^ CD8^+^ T cells (**B**), %Granzyme^+^ IFNγ^+^ CD8^+^ T cells (**C**), and %Perforin^+^ IFNγ^+^ CD8^+^ T cells (**D**) in unstimulated and individual APOB mesopool-stimulated peripheral blood mononuclear cells (PBMCs). **E**, Frequency of %TNF^+^ IFNγ^+^ CD8^+^ T cells in unstimulated and individual APOB mesopool-stimulated PBMCs. Data from 8 independent donors. Paired statistical comparisons (**E**) between unstimulated and stimulated groups were performed using the Wilcoxon matched-pairs signed-rank test. Numerical *P* values are listed at the top of each graph. Data are represented as mean±SEM.

Next, we used the ICS assay (schematic in Figure 3A) to further characterize the functionality of APOB-reactive activated CD8^+^ T cells. T-cell receptor-mediated activation results in the expression of cytotoxic and inflammatory cytokines in CD8^+^ T cells. Recent single-cell (sc)RNA sequencing of anti-CD3/CD28-stimulated and control unstimulated human T cells from blood and various tissue sites has defined the heterogeneity of the molecular programs associated with resting and activated T cells.^32^ While CD8^+^ T cells can express the cytotoxic gene module in both the resting and activated states, the cytokine gene module appears specifically in the highly activated CD8^+^ T cells, which consistently expressed IFNγ across tissue sites.

The ICS assay confirmed coexpression of IFNγ and TNF in APOB-reactive CD8^+^ T cells (Figure 3B and 3E). All IFNγ+ cells also co-expressed granzyme B (Figure 3C; Figure S3A, top) and perforin (Figure 3D; Figure S3A, bottom). Thus, HLA class I-restricted human APOB-derived peptides elicit a strong IFNγ response in CD8^+^ T cells. Coexpression of granzyme B and perforin suggests that APOB-reactive activated CD8^+^ T cells possess both inflammatory and cytotoxic potential.

### Identification of Dominant HLA-A*02:01-Restricted APOB Epitopes

**Figure 4.**
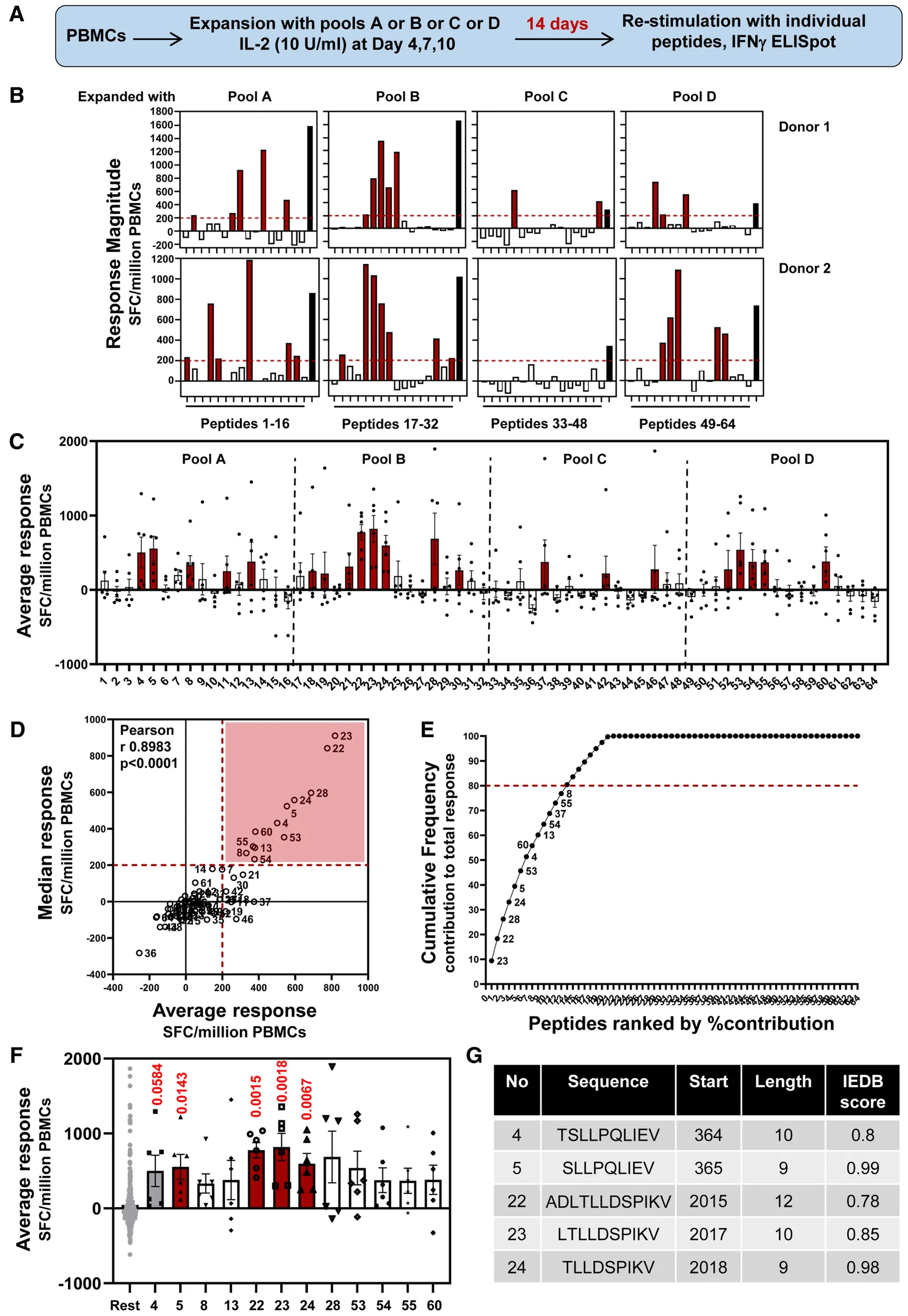
Identification of human leukocyte antigen (HLA)-A*02:01-restricted dominant human APOB epitopes. A, Schematic workflow for deconvolution of cytokine responses to individual HLA-A*02:01-restricted APOB peptides using an IFNγ ELISpot assay. **B**, Representative interferon-gamma (IFNγ) ELISpot data from 2 donors. Responses to individual APOB mesopools A to D (*X* axes) were measured as spot-forming cells (SFCs) per million peripheral blood mononuclear cells (PBMCs; *Y* axes). Background-subtracted (responses in stimulated minus unstimulated sets) frequencies are shown. Red dotted lines depict a threshold of 200 SFCs/million PBMCs. Response values that were below or above the threshold are shown in white and red, respectively. Black bars depict responses to the total mesopool. **C**, Background- subtracted SFCs/million PBMCs (mean±SEM) for each of the 64 APOB-derived peptides. N=6 independent donors. **D**, Scatter plots showing Pearson correlation (*r*) between average (*X* axis) and median (*Y* axis) responses (SFCs/million PBMCs) to the 64 APOB class I epitopes. Background-subtracted values are plotted. Black solid lines and red dotted lines correspond to zero and threshold (200) values, respectively. Red shaded area: peptides with >200 mean and median SFCs/million PBMCs (n=6). **E**, APOB peptides were ranked from 1 to 64 (*X* axis) based on their percent contribution (Y-axis) to the total APOB-specific response as detected in the ELISpot assay (n=6 donors). The serial numbers (from Table S1) of the 13 peptides that accounted for 80% of the total response (indicated as a red dotted line) are labeled on the plot. **F**, Responses (mean±SEM) to individual peptides P4 (gray), 5, 8, 13, 22, 23, 24, 28, 53, 54, 55, and 60 (red: significantly higher; white: not significant) and to the remaining peptides (black). **G**, Table showing sequences, start sites, lengths, and Immune Epitope Database (IEDB) score of the dominant APOB epitopes (red and gray bars in F). Statistical tests (**F**) were performed using the Kruskal-Wallis test with Dunn multiple-comparison adjustment. Numerical *P* values are listed at the top of each graph.

In this study, all 4 mesopools (A–D) induced IFNγ expression over unstimulated controls (Figure 3B through 3E; Figure S3A) in the ICS assay. Among them, mesopool B (APOB P17–P32) elicited the highest frequency of IFNγ-producing CD8^+^ T cells, followed by mesopools A, D, and C (Figure S3B). This likely reflects preferential recognition of dominant epitopes by APOB-reactive CD8^+^ T cells in multiple donors. We had previously optimized an expansion-based IFNγ ELISpot assay to identify class II-restricted CD4^+^ T-cell-activating dominant epitopes in APOB.^6^ Here, we first confirmed that this assay can also be utilized to deconvolve the class I-restricted APOB peptide pools (Figure S3C). The IFNγ ELISpot assay detected positive responses in stimulated PBMCs over the unstimulated control, which were inhibited by a blocking monoclonal antibody to HLA class I but remained unaffected by class II blocking (Figure S3C; Table S2). To dissect responses to individual peptides, we expanded PBMCs from HLA-A*02:01+ donors using APOB mesopools and then restimulated them with each peptide in that mesopool (schematic in Figure 4A). SFCs were counted, and average spots from replicate wells were determined. Response magnitude (SFCs per million PBMCs) was estimated after subtracting background spot counts from control unstimulated wells. Positive (red bars, Figure 4B and 4C) and negative (white bars, Figure 4B and 4C) epitopes were scored based on a threshold of 200 peptide-induced SFCs/million PBMCs. Total responses to each mesopool A to D (black bars, Figure 4B) served as positive controls. Several peptides elicited strong IFNγ responses, particularly those in mesopool B (Figure 4B, representative responses in 2 donors; Figure 4C, mean responses in 6 donors). We identified 21 positive epitopes with average responses >200 SFCs/million PBMCs (red bars, Figure 4C). Half (8 of 16) of the peptides in mesopool B elicited positive responses compared with ≈30% in mesopools A and D and ≈20% in mesopool C (Figure 4C). The magnitude of the total response was highest against the peptides in mesopool B (mean SFCs/million PBMCs: Pool A, 2508.64; Pool B, 4363.09; Pool C, 365.08; Pool D, 904). To evaluate response robustness, we compared mean and median responses for each epitope and observed a strong positive correlation (Pearson *r* =0.8983; *P*<0.0001; Figure 4D). Thus, both response strength and breadth (Figure 4C) were highest for peptides in mesopool B (P17_1340__–1348_ to P32_2356__–2365_) and lowest for mesopool C (P33_2376__–2384_ to P48_3734__–3743_).

As median values are less sensitive to skewing than the mean, we compared the correlation between the mean and median responses to each epitope. Twelve peptides triggered consistently strong responses across all donors, with both mean and median responses >200 SFCs/million PBMCs (red shaded area, Figure 4D). These epitopes accounted for 80% of the total response (Figure 4E). Four peptides, P5, P22, P23, and P24, induced significantly stronger responses than the remaining peptides (red bars, Figure 4F). Peptide P4 also elicited a strong response (gray bar, Figure 4F). These 5 peptides (P4_364__–373_, P5_365__–373_, P22_2015__–2026_, P23_2017__–2026_, and P24_2018__–2026_) mapped to just 2 sites in human APOB (Figure 4G). These findings reveal a previously underappreciated dimension of autoreactivity in atherosclerosis and open new avenues for antigen-specific immune monitoring and therapeutic targeting of CD8^+^ T cells.

### HLA-A*02:01-Restricted APOB-Reactive CD8^+^ T Cells in Patients With Severe CAD

**Figure 5.**
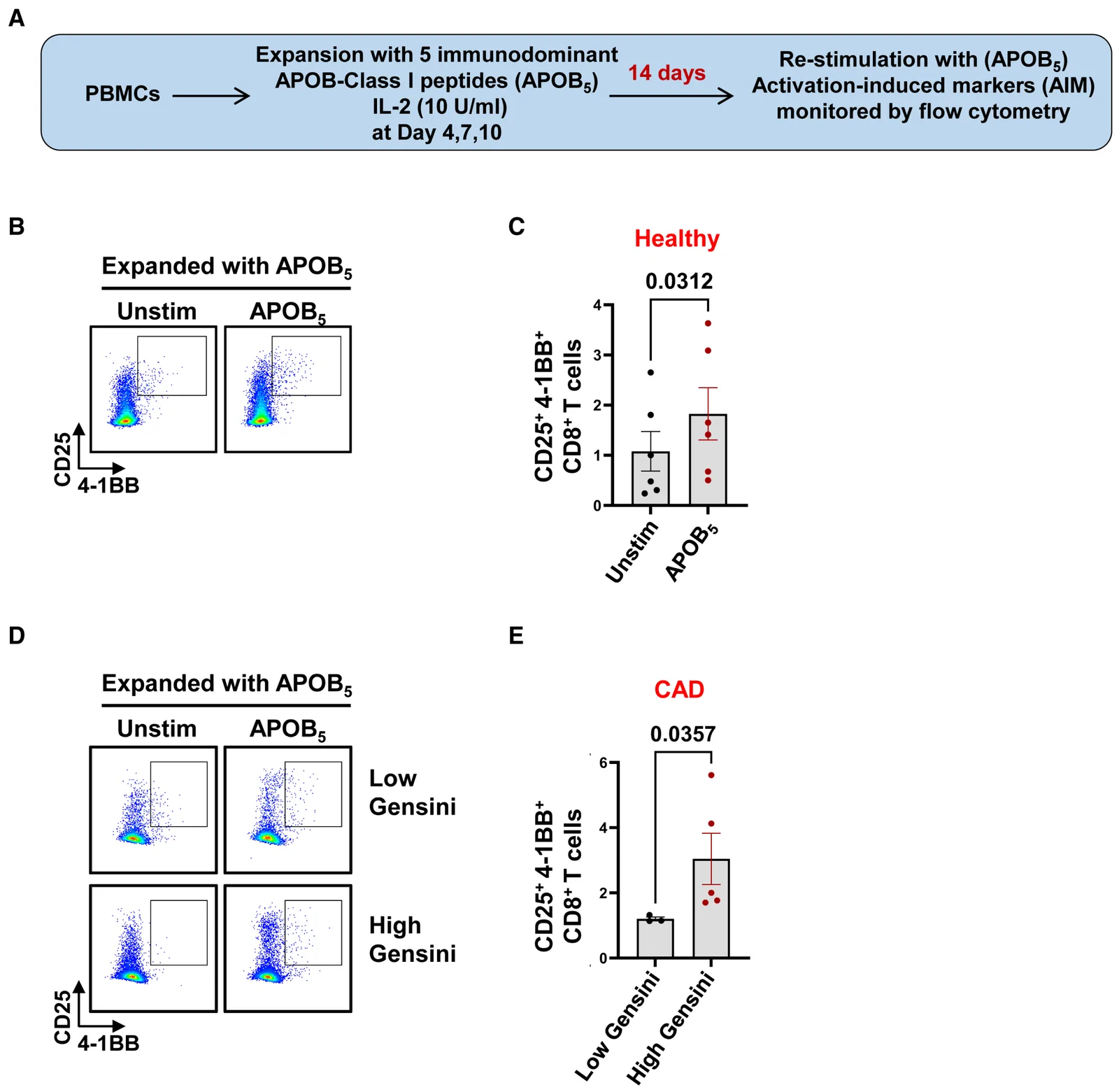
Human leukocyte antigen (HLA)-A*02:01-restricted APOB-reactive CD8+ T cells in healthy subjects and patients with coronary artery disease (CAD) of varied severity. A, Schematic diagram depicting the workflow for expansion and subsequent restimulation of human peripheral blood mononuclear cells (PBMCs) with a pool of 5 immunodominant human APOB-derived HLA-A*02:01-restricted peptides (APOB_5_). Antigen-dependent expression of T cell activation markers was examined using flow cytometry. **B**, Representative flow cytometry plots showing CD25^+^4-1BB^+^ CD8^+^ T cells in unstimulated and APOB_5_-stimulated PBMCs in healthy subjects. **C**, Fold change in the frequency of %CD25^+^4-1BB^+^ CD8^+^ T cells in APOB_5_-stimulated vs unstimulated PBMCs in healthy subjects. The value nearest to the median of % CD25^+^4-1BB^+^ CD8^+^ T cells in unstimulated controls was scaled to 1 and all the other values were plotted as fold change with respect to it. N=6 independent healthy donors. **D**, Representative flow cytometry plots showing CD25^+^4-1BB^+^ CD8^+^ T cells in unstimulated and APOB_5_-stimulated PBMCs in patients with no/mild CAD (low Gensini) or severe CAD (high Gensini). **E**, Fold change in the frequency of %CD25^+^4-1BB^+^ CD8^+^ T cells in APOB_5_-stimulated vs unstimulated PBMCs in patients with no/mild CAD (low Gensini) or severe CAD (high Gensini). The frequency of CD25^+^4-1BB^+^ CD8^+^ T cells in unstimulated cells from each donor was normalized to 1. N=3 patients in the low Gensini group and N=5 patients in the high Gensini group. Paired statistical comparisons (**C**) between unstimulated and stimulated groups were performed using the Wilcoxon matched-pairs signed-rank test. Unpaired statistical comparisons in (**E**) were done using the Mann-Whitney *U* test. Numerical *P* values are listed at the top of each graph. Data are represented as mean±SEM.

Having identified the 5 immunodominant HLA-A*02:01-restricted APOB epitopes (P4, P5, P22, P23, P24), we examined CD8^+^ T-cell responses to these epitopes by the expansion- and restimulation-based AIM assay (schematic in Figure 5A). In 6 healthy donors, a significant increase in CD25^+^4-1BB^+^ CD8^+^ T cells was observed upon restimulation with a pool of these 5 peptides (APOB_5_) compared with unstimulated controls (Figure 5B and 5C). As a positive control, we stimulated and expanded healthy PBMCs with CEFX-I (JPT Peptide Technologies), a pool of 80 known peptide epitopes for a broad range of HLA class I subtypes and different infectious agents (schematic in Figure S4A). Restimulating these cells with CEFX-I elicited a strong increase in the responder cells (CD25^+^4-1BB^+^CD8^+^ T cells; Figure S4B and S4C). Restimulation of CEFX-I-expanded PBMCs with APOB_5_ did not elicit a response as compared with unstimulated controls (Figure S4B and S4C). Thus, the CD8^+^ T-cell response to the 5 immunodominant APOB epitopes is specific.

To examine APOB_5_-specific responses in patients with CAD, we analyzed clinical samples from the Coronary Assessment in Virginia cohort. We compared CD8^+^ T-cell responses to APOB_5_ in matched clinical samples (Table S3) from patients with no or low CAD versus high CAD, as assessed by angiographic disease burden, expressed as Gensini scores.^33^ Restimulation of PBMCs with the APOB_5_ pool triggered a significantly higher increase in CD25^+^4-1BB^+^ CD8^+^ T cells in patients with high CAD (median Gensini score, 75), as compared with patients with low CAD (median Gensini score, 0; Figure 5D and 5E). In a second cohort, we measured APOB_5_-specific CD8^+^ T-cell responses in PBMCs from 6 patients undergoing surgical carotid or femoral endarterectomy (schematic in Figure S4D and clinical details in Table S4). We observed a significant increase in CD25^+^4-1BB^+^ CD8^+^ T cells in these patients upon restimulation with APOB_5_ (Figure S4E and S4F).

## Discussion

This is the first report of a systematic deconvolution of CD8^+^ T-cell responses to all putative HLA-A*02:01-restricted epitopes in human APOB, a well-known atherosclerosis-related autoantigen with established clinical relevance. Our key findings include: (1) the discovery of >60 potential epitopes spanning the human APOB protein using in silico prediction tools, (2) characterization of effector memory markers and the T-cell activation-related proteins CD69, CD25, and 4-1BB on APOB-specific CD8^+^ T cells in adult human blood, (3) expression of cytotoxic molecules (GZMB, perforin) and inflammatory cytokines (IFNγ and TNF) by CD8^+^ T cells in response to APOB peptides, (4) serial deconvolution of cytokine responses and identification of 2 dominant antigenic sites in APOB, and (5) significantly increased APOB-reactive CD8^+^ T cells in patients with severe CAD.

Despite abundant data showing the presence of cytotoxic CD8^+^ T cells in plaque and the periphery of preclinical mouse models and humans, their role and antigen specificity remain unclear. One study found that *Apoe*^−^^/−^ mice developed atherosclerosis both in wild type (WT) and CD8-knockout backgrounds.^34^ Two other studies depleted CD8^+^ T cells using anti-CD8 antibodies in *Ldlr*^−^^*/*−^ and *Apoe*^−^^*/*−^ mice, demonstrating a reduction in disease severity.^35,36^ Hence, there is a critical need to develop tools for the detection of ApoB-specific CD8^+^ T cells in humans and to further understand their role in disease development.

Previous studies identified MHC-I-restricted autoreactive CD8^+^ T cells targeting a single APOB peptide, p210, in the spleen of wild-type and *Apoe*^−^^*/*−^ atherosclerotic mice.^37^ Several studies examined CD8^+^ T-cell responses to human APOB-derived peptides presented by the HLA-A*02:01 allele.^38–40^ Two studies used immunized transgenic mice expressing human APOB and the HLA-A*02:01 allele,^39,40^ while another study utilized an HLA-A*0201 pentamer to detect P210-specific CD8^+^ T cells in human PBMCs.^38^ None of these previous studies compared the antigenic potential of individual APOB-derived peptides in healthy or atherosclerotic human subjects. Since not all peptides that can bind HLA class I alleles can activate CD8^+^ T cells, evaluating responses to epitopes in HLA-typed donors remains essential. Indeed, in our analyses, we identified 5 peptides that map to 2 specific regions in human APOB. These peptides significantly elicit a high number of SFCs in the IFN-γ ELISpot assay. Phenotypically, APOB-reactive CD8^+^ T cells identified in our study resembled cytotoxic and inflammatory effector memory CD8^+^ T cells. Such cells were previously reported in blood and plaque lesions from atherosclerotic patients, but their antigen specificity was unknown.^29–31^ Presentation of 2 of these ApoB HLA-A*02:01-associated peptides (P4 and P24 in Figure 4G) on human MHC-I was independently confirmed by immunopeptidomics.^41^

In addition to the presence of APOB-reactive CD8^+^ T cells in healthy individuals, we also observed these cells in 2 independent cohorts of patients with varied severity of CAD or those undergoing endarterectomy. A higher CD8^+^ T-cell response to APOB peptides was observed in patients with severe CAD than no or mild CAD, showing that APOB-reactive CD8+ T cells are associated with disease progression. Previous studies have shown the presence of autoreactive CD8^+^ T cells in patients with type 1 diabetes.^9,42,43^ More recently, in situ immunofluorescence staining using preproinsulin-specific tetramers demonstrated the presence of autoreactive CD8^+^ T cells within pancreatic tissue sections obtained from individuals with diabetes and nondiabetic controls.^44^

The findings from this study collectively reconcile previous observations that utilized scRNA (single cell RNA)- and TCR-sequencing (Seq) technologies showing expansion and activation of CD8^+^ T cells in mouse and human plaques.^3,4^ However, the specific plaque antigens recognized by these expanded cells were previously unknown. Molecular mimicry has recently been proposed as a possible source of autoreactivity in human atherosclerosis. For instance, influenza-specific T-cell receptors cross-reacted with 2 self-proteins expressed by endothelial cells and smooth muscle cells.^29^ Another study showed that 1 CD8^+^ T-cell clone specific for CMV expanded in both blood and carotid plaque.^4^ Furthermore, analysis of the immunopeptidome of carotid plaques showed 2 potential peptides related to CMV and *C. pneumoniae*. However, these viral peptides are indistinguishable from peptides from self-proteins.^45^

In conclusion, using systematic screening via in silico and in vitro approaches, our work collectively documents the presence of ApoB-specific CD8^+^ T cells in the periphery of healthy human adults, which further confirms the autoimmune nature of atherosclerosis. We also demonstrated that these 5 immunodominant HLA-A*02:01-restricted ApoB peptides are suitable for monitoring CD8^+^ T-cell autoreactivity in patients with CAD, highlighting their relevance in clinical atherosclerosis.

## ARTICLE INFORMATION

### Acknowledgments

The authors thank the Augusta University(AU) Georgia Cancer Center Flow and Mass Cytometry Core Facility (RRID: SCR_025747) and the Flow Cytometry Core at the La Jolla Institute for Immunology for the acquisition of flow cytometry data. The authors also thank Clinical Core at the La Jolla Institute for Immunology for the collection of human blood samples. The authors acknowledge the support and contribution of the Immune Monitoring Shared Resource at the Georgia Cancer Center, Augusta University (RRID: SCR_026590), for facilitating human leukocyte antigen (HLA) typing. The authors also thank the members of the vascular surgery team at the Medical College of Georgia at AU and the members of the cardiac catheterization laboratories at the University of Virginia (UVA) for facilitating the collection of the patient samples. The graphical abstract was generated using graphics from Servier Medical Art.

### Disclosures

K. Ley is the founder and coowner of Atherovax, Inc. He receives no compensation from Atherovax. No Atherovax funds were used in this study. K. Ley and P. Roy are named as coinventors on a patent application (provisional application no. 63/789 764, filed by the La Jolla Institute for Immunology; approval status pending) that is related to the use of human APOB epitopes and related methods for modulating inflammatory responses and treating adverse cardiovascular events, disease, and atherosclerosis. The other authors report no conflicts.

### Supplemental Material

Tables S1–S4

Figures S1–S4

Major Resources Table

## Supplementary Material
