# Supplementary material for: Increased Autoreactive CD8+ T Cells to Apolipoprotein B Epitopes in Patients With Severe Coronary Artery Disease
Source: Circ Res. 2026 Jul 23;139(6):e326804. doi: 10.1161/CIRCRESAHA.125.326804 (PMC13426068; doi:10.1161/CIRCRESAHA.125.326804)
Supplement: Supplementary file 1 [file res-139-e326804-s001.pdf]

Supplemental Material for

Increased autoreactive CD8+ T cells to apolipoprotein B epitopes in patients with severe coronary artery disease

Payel Roy, PhD[1,2,9]†, Smriti Parashar, PhD[1]†, John Sidney, PhD[3], Megh Mehta, BS[1], Ishita Tandon, PhD[1], Margaret Beddall, ALM[1], Yan Wang, PhD[1], Irina Baranovskaya, PhD[1], Andrew Soo Hoo, MD[4], Ralph Ierardi, MD[4], Gautam Agarwal, MD[4], William D. Jordan, MD[4], Coleen A. McNamara, MD[5], Alessandro Sette, PhD[3,6], Hossam Abdelsamed, PhD[1,7*], Klaus Ley, MD[1,2,7,8*#]

[1] Immunology Center of Georgia, Augusta University, 1410 Laney Walker Blvd, CN2119, Augusta, GA 30912

[2] Center for Inflammation Biology, La Jolla Institute for Immunology, 9420 Athena Circle Drive, La Jolla, CA 92037

[3] Center for Infectious Disease and Vaccine Research, La Jolla Institute for Immunology, 9420 Athena Circle Drive, La Jolla, CA 92037

[4] Department of Surgery, Medical College of Georgia at Augusta University, Augusta, GA 30912

[5] Department of Medicine/Cardiovascular Division and Carter Immunology Center, University of Virginia, Charlottesville, VA, USA.

[6] Department of Medicine, University of California, San Diego, La Jolla, CA 92093

[7] Department of Physiology, Medical College of Georgia at Augusta University, Augusta, GA 30912

[8] Department of Bioengineering, University of California San Diego, 9500 Gilman Drive, La Jolla, CA, 92093 USA

[9] Present affiliation: Department of Biochemistry, Indian Institute of Science, Bengaluru, India

† Contributed equally   * Co-corresponding author

Lead contact: Klaus Ley, kley@augusta.edu

Contents of Supplement

Table S1-S4

Figures S1-S4

Major resources table

Table S1: HLA-A*0201 binding human APOB peptides

Peptide Number	start	end	length	peptide	9-mer core	IEDB score	rank
1	10	18	9	ALLALPALL	ALLALPALL	0.836847	0.09
2	142	153	12	FLYPEKDEPTYI	FLYDEPTYI	0.931697	0.04
3	357	366	10	GLSDEAVTSL	GLDEAVTSL	0.76559	0.14
4	364	373	10	TSLLPQLIEV	TLLPQLIEV	0.802248	0.11
5	365	373	9	SLLPQLIEV	SLLPQLIEV	0.992869	0.01
6	406	414	9	LLIDVVTYL	LLIDVVTYL	0.991445	0.01
7	406	415	10	LLIDVVTYLV	LLIDVVTYV	0.791125	0.12
8	441	449	9	TLYALSHAV	TLYALSHAV	0.900823	0.04
9	580	589	10	ILPWEQNEQV	ILWEQNEQV	0.80776	0.1
10	592	600	9	FVASHIANI	FVASHIANI	0.836352	0.09
11	647	655	9	SLDPASAKI	SLDPASAKI	0.82452	0.1
12	659	667	9	LIFDPNNYL	LIFDPNNYL	0.794594	0.11
13	829	838	10	YIFMENAFEL	YIFENAFEL	0.742999	0.16
14	934	942	9	KLLSGGNTL	KLLSGGNTL	0.775515	0.13
15	1028	1036	9	ALVDTLKFV	ALVDTLKFV	0.946944	0.03
16	1250	1258	9	TLQDHLNSL	TLQDHLNSL	0.867318	0.07
17	1340	1348	9	FTIPKLYQL	FTIPKLYQL	0.816479	0.1
18	1354	1362	9	GVLDLSTNV	GVLDLSTNV	0.789515	0.12
19	1867	1875	9	RLNTDIAGL	RLNTDIAGL	0.775078	0.13
20	1899	1907	9	VMAPFTMTI	VMAPFTMTI	0.945987	0.03
21	1931	1939	9	FLLKAEPLA	FLLKAEPLA	0.778996	0.13
22	2015	2026	12	ADLTLLDSPIKV	ALLDSPIKV	0.783392	0.13
23	2017	2026	10	LTLLDSPIKV	LLLDSPIKV	0.84936	0.08
24	2018	2026	9	TLLDSPIKV	TLLDSPIKV	0.98341	0.01
25	2028	2036	9	LLLSEPINI	LLLSEPINI	0.916929	0.04
26	2177	2185	9	YMIQFDQYI	YMIQFDQYI	0.932441	0.04
27	2201	2209	9	IIDEIIEKL	IIDEIIEKL	0.898399	0.04
28	2211	2220	10	SLDEHYHIRV	SLDEYHIRV	0.726944	0.17
29	2309	2317	9	RINDILEHV	RINDILEHV	0.91229	0.04
30	2355	2364	10	QVLMDKLVEL	QLMDKLVEL	0.775655	0.13
31	2356	2364	9	VLMDKLVEL	VLMDKLVEL	0.995866	0.01
32	2356	2365	10	VLMDKLVELA	VLMDKLVEA	0.838608	0.09
33	2376	2384	9	KLSNVLQQV	KLSNVLQQV	0.946947	0.03
34	2396	2404	9	FIDDAVKKL	FIDDAVKKL	0.760766	0.14
35	2419	2427	9	FLDMLIKKL	FLDMLIKKL	0.810587	0.1
36	2449	2457	9	RLNGEIQAL	RLNGEIQAL	0.84668	0.08
37	2469	2477	9	FLEETKATV	FLEETKATV	0.865789	0.07
38	2483	2491	9	SLQDTKITL	SLQDTKITL	0.772214	0.13
39	2524	2532	9	YQMDIQQEL	YQMDIQQEL	0.932683	0.04
40	2578	2587	10	KALVEQGFTV	KLVEQGFTV	0.727475	0.17
41	2579	2587	9	ALVEQGFTV	ALVEQGFTV	0.979995	0.01
42	2725	2733	9	TLNLNDFQV	TLNLNDFQV	0.761049	0.14
43	3070	3078	9	FLNNYALFL	FLNNYALFL	0.961079	0.03
44	3077	3085	9	FLSPSAQQA	FLSPSAQQA	0.722965	0.17
45	3531	3544	14	KLQGTSKIDDIWNL	KLIDDIWNL	0.800153	0.11
46	3671	3679	9	FLKNIILPV	FLKNIILPV	0.783702	0.13
47	3687	3696	10	FLKLDVTTSI	FLLDVTTSI	0.812076	0.1
48	3734	3743	10	GLKLNDLNSV	GLLNDLNSV	0.755279	0.14
49	3736	3744	9	KLNDLNSVL	KLNDLNSVL	0.821462	0.1
50	3736	3745	10	KLNDLNSVLV	KLNDLNVLV	0.801536	0.11
51	3743	3751	9	VLVMPTFHV	VLVMPTFHV	0.744059	0.16
52	3780	3789	10	ALNLPTLPEV	ALLPTLPEV	0.854729	0.08
53	3806	3815	10	SLIPFFEITV	SLIPFEITV	0.823349	0.1
54	4166	4178	13	GLKDNVFDGLVRV	GLFDGLVRV	0.931128	0.04
55	4170	4178	9	NVFDGLVRV	NVFDGLVRV	0.937023	0.03
56	4188	4197	10	HLIDSLIDFL	HLIDSLIFL	0.759155	0.14
57	4298	4306	9	NLQDLLQFI	NLQDLLQFI	0.760649	0.14
58	4324	4336	13	YLINYIQDEINTI	YLIDEINTI	0.776662	0.13
59	4328	4336	9	YIQDEINTI	YIQDEINTI	0.866871	0.07
60	4335	4344	10	TIFSDYIPYV	TIFDYIPYV	0.767466	0.14
61	4401	4410	10	YELEEKIVSL	YLEEKIVSL	0.740937	0.16
62	4498	4506	9	KLQDFSDQL	KLQDFSDQL	0.880928	0.06
63	4531	4539	9	FLIYITELL	FLIYITELL	0.862895	0.07
64	4553	4561	9	KLAPGELTI	KLAPGELTI	0.894725	0.05

Table S1: HLA-A*0201 binding human APOB peptides. Details of 64 HLA-A*0201 binding human APOB peptides used in this study.

Table S2: IFNγ responses to APOB class-I peptides in the presence of HLA blocking antibodies

Donor	Pool	NO BLOCK	Class I_BLOCK	Class II_BLOCK
1	A	1487	310	2013
1	B	1623	540	1563
1	C	498	70	735
1	D	638	147	672
2	A	383	92.5	698
2	B	918	335	1370
2	C	855	12.5	438
2	D	335	-295	685
3	A	1588	1205	1413
3	B	1660	1508	487.5
3	C	298	-45	-30
3	D	385	85	218

Table S2: IFNγ responses to APOB class-I peptides in the presence of HLA blocking antibodies. PBMCs from 3 healthy donors were each expanded with mesopool A-D separately. Each expanded set was restimulated with the cognate mesopool in the presence or absence of the indicated blocking antibodies and analyzed by ELISpot. Table shows IFNγ responses to APOB class-I peptides, either alone or in the presence of pan-HLA-I or pan-HLA-II (DP+DQ+DR) blocking antibodies. Background subtracted values are shown. Related to Figure S3C.

Table S3: Demographic and Clinical Parameters for CAD patients from CAVA cohort

Demographics	Mild/Low CAD (n=3) Variable Count [%] or Mean [± SEM]	High CAD (n=5) Variable Count [%] or Mean [± SEM]
Age (years)	60 [± 2.8]	59.8 [± 2.6]
Sex (Male)	3(100%)	5(100%)
Race (White)	3 (100%)	5(100%)
Ethnicity (Non-Hispanic)	3(100%)	5(100%)
Gensini score (median)	0	75
Diabetes	2 (66.66%)	3(60%)
Hypertension	2 (66.66%)	3(60%)
Current smoker	1 (33.3%)	2(40%)
Former smoker	1 (33.3%)	2(40%)
SBP, systolic blood pressure (mmHg)	133.3 [± 4.9]	132[± 5.5]
DBP, diastolic blood pressure (mmHg)	76.3 [± 3.7]	77.4[± 7.2]
BMI (kg/m2)	43.5 [± 4.2]	32.8[± 1.4]
**Medications**		
Statins (Yes)	1 (33.3%)	4(80%)
Diuretics (Yes)	1 (33.3%)	1(20%)
Beta Blockers (Yes)	3(100%)	4(80%)
Calcium Channel Blockers (Yes)	1 (33.3%)	0(0%)
ACEi, Angiotensin converting enzyme inhibitors (Yes)	0 (0%)	1(20%)
NSAID (Yes)	2 (66.6%)	5(100%)
**Labs**		
Cholesterol (mg/dL)	150 [± 14]	127.4[± 15.5]
HDL (mg/dL)	39.3 [± 2.5]	30.6[± 2.3]
LDL-C (mg/dL)	86.6 [± 10.5]	72.2[± 12.8]
POC (Point-of-care) Glucose (mg/dL)	108.3 [± 3.6]	114.8[± 11.2]
HbA1c (%)	6.5 [± 0.3]	6.6[± 0.4]

Table S3: Demographic and Clinical Parameters for CAD patients from CAVA cohort. Clinical table summarizing details related to demographics, medications, lab values and Gensini scores of patients in the CAVA cohort used for this study. Categorical variables are plotted as counts and percentages, while continuous variables are shown as mean ± SEM within the cohort (sample size = 3 patients for mild/low CAD and 5 patients for high CAD).

Table S4: Demographic and Clinical Parameters for endarterectomy patients

**Demographics**	**Variable Count [%] or Mean [± SEM]**
Age (years)	65.7 [± 2.3]
Sex (Male)	4 (66.7%)
Race (White)	6 (100%)
Ethnicity (Non-Hispanic)	6 (100%)
Endarterectomy (% Carotid)	5 (83.3%)
Diabetes	3 (50%)
Hypertension	6 (100%)
Hyperlipidemia	5 (83.3%)
Current smoker	2 (33.3%)
Former smoker	4 (66.7%)
SBP, systolic blood pressure (mmHg)	148.3 [± 6.3]
DBP, diastolic blood pressure (mmHg)	76.3 [± 6.2]
BMI (kg/m2)	25.7 [± 1.9]
**Medications**	
Statins (Yes)	6 (100%)
Diuretics (Yes)	0 (0%)
Beta Blockers (Yes)	2 (33.3%)
Calcium Channel Blockers (Yes)	1 (16.7%)
ACEi, Angiotensin converting enzyme inhibitors (Yes)	3 (50%)
ARB, Angiotensin receptor blockers (Yes)	0 (0%)
NSAID (Yes)	0 (0%)
Acetaminophen (Yes)	2 (33.3%)
**Labs**	
WBC (x1000 cells/uL)	8.9 [± 1.2]
Hemoglobin (g/dL)	10.7 [± 0.7]
Hematocrit (%)	31 [± 2.6]
Platelets (x100k)	191.8 [± 21.2]
Cholesterol (mg/dL)	138.4 [± 12.9]
HDL (mg/dL)	45.8 [± 5]
LDL-C (mg/dL)	63.4 [± 9.4]
POC (Point-of-care) Glucose (mg/dL)	108.8 [± 12.5]
HbA1c (%)	6.4 [± 0.3]

Table S4: Demographic and Clinical Parameters for endarterectomy patients. Clinical table summarizing details related to demographics, medications, lab values and site of endarterectomy of patients in the AU patient cohort used for this study. Categorical variables are plotted as counts and percentages, while continuous variables are shown as mean ± SEM within the cohort (sample size = 6 patients).

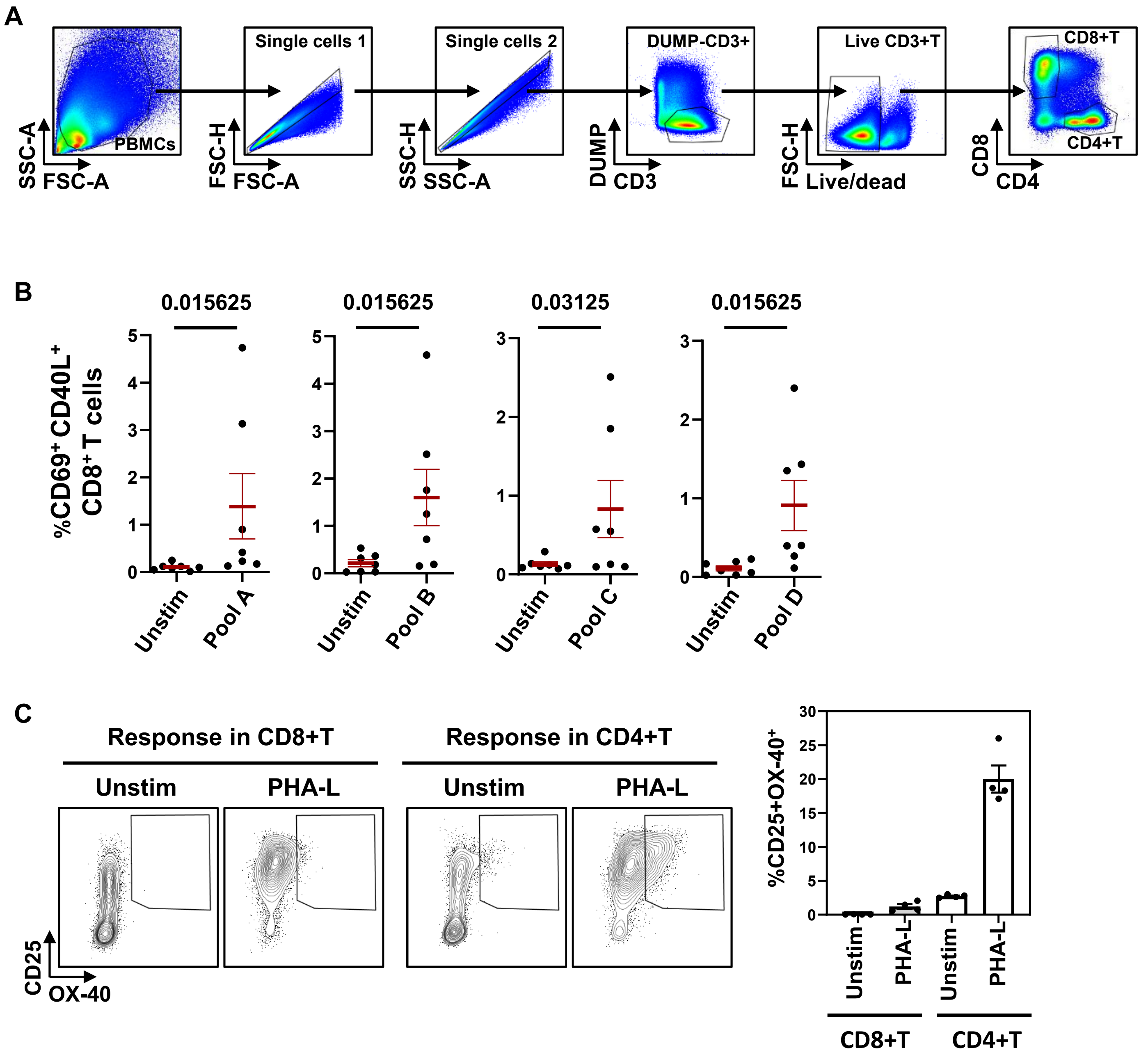
A
Single cells 1
Single cells 2
DUMP-CD3+
Live CD3+T
CD8+T
CD4+T
PBMCs
SSC-A
FSC-A
FSC-H
FSC-A
SSC-H
SSC-A
DUMP
CD3
FSC-H
Live/dead
CD8
CD4
B
0.015625
0.015625
0.03125
0.015625
%CD69+ CD40L+ CD8+ T cells
Unstim
Pool A
Unstim
Pool B
Unstim
Pool C
Unstim
Pool D
C
Response in CD8+T
Response in CD4+T
Unstim
PHA-L
Unstim
PHA-L
CD25
OX-40
%CD25+OX-40+
Unstim
PHA-L
Unstim
PHA-L
CD8+T
CD4+T

Figure S1

Figure S1: Controls in an AIM assay workflow to evaluate human CD8^{+}T responses to APOB (A) Gating strategy in flow cytometry to designate CD8^{+} or CD4^{+} T cells for AIM marker evaluation **(B)** Frequencies (mean ± SEM) of %CD69^{+}CD40L^{+} CD8^{+}T cells in unstimulated and individual mesopool stimulated PBMCs. N=7 independent donors **(C)** Representative flow cytometry plots showing expression of CD25^{+}OX-40^{+} in CD8^{+} and CD4^{+} T cells in response to polyclonal stimulation PHA-L (left). Bar-graph showing %CD25^{+}OX-40^{+} (right). Data from four independent mesopool-stimulated sets in one donor (C). Pairwise comparisons between unstimulated and stimulated sets (B) were done with Wilcoxon matched-pairs signed rank test. Numerical p values are listed at the top of each graph. Data represented as mean ± SEM.

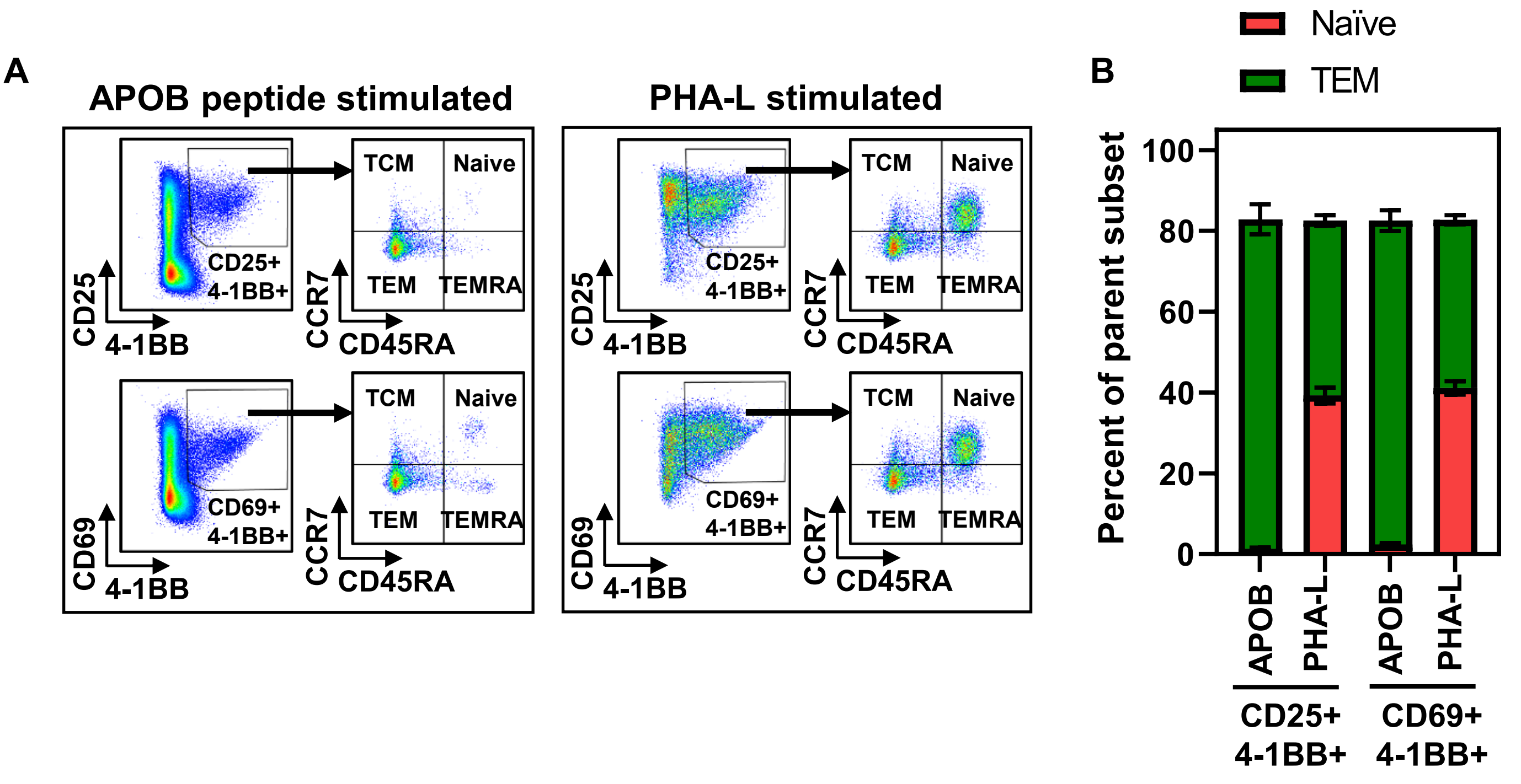

Figure S2: APOB-peptide stimulation enriches for TEM CD8⁺ T cell subsets with upregulation of AIM markers (A) Representative flow cytometry plots showing enrichment of TEM subset with upregulation of AIM markers following APOB peptide stimulation compared to polyclonal PHA-L stimulation. **(B)** Bar graph showing frequency of naïve (red) and TEM (green) subsets upregulating AIM markers following APOB versus PHA-L stimulation.

Figure S2

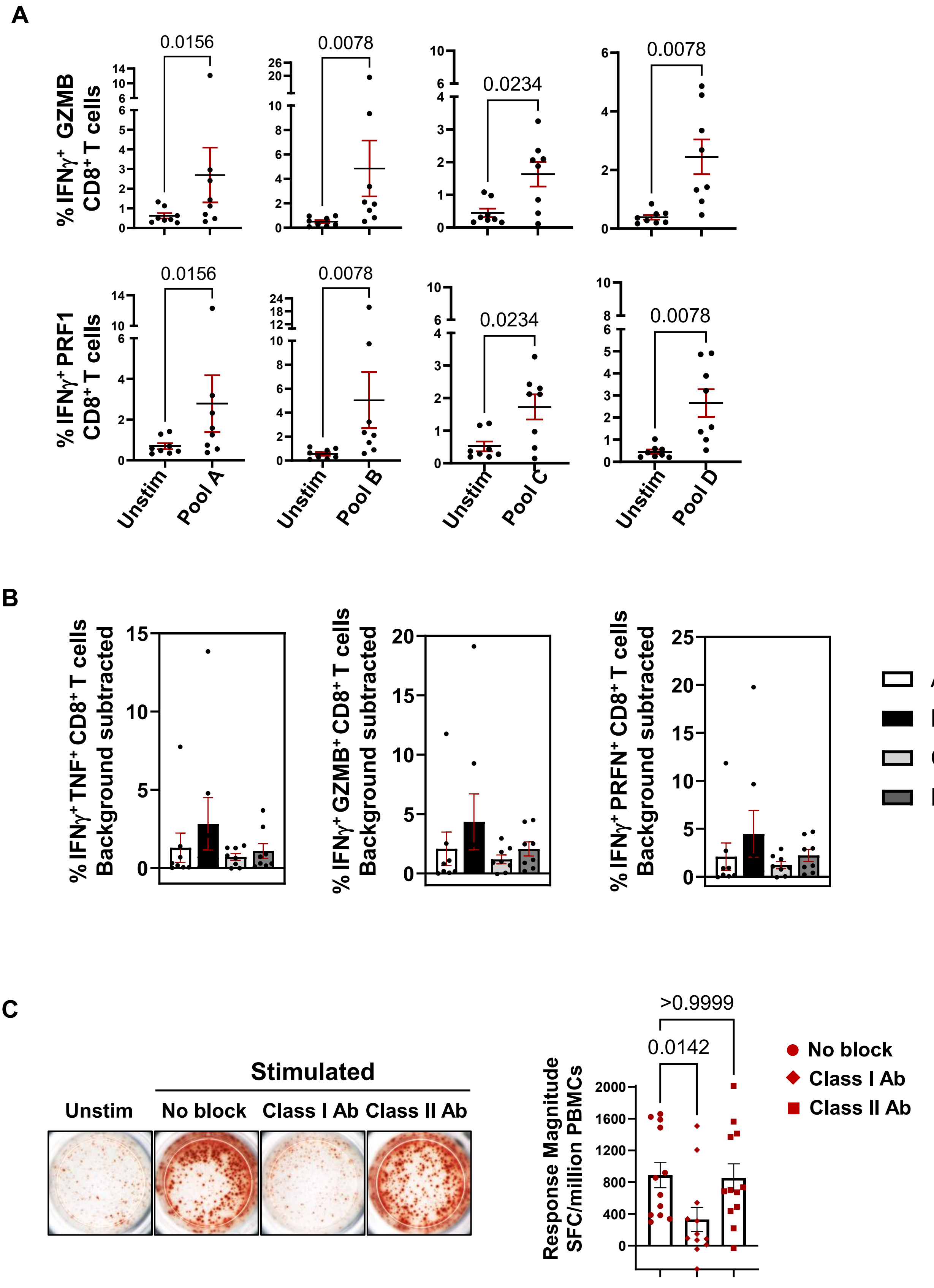
A
% IFNγ+ GZMB CD8+ T cells
% IFNγ+ PRF1 CD8+ T cells
0.0156
0.0078
0.0234
0.0078
Unstim
Pool A
Pool B
Pool C
Pool D
B
% IFNγ+ TNF+ CD8+ T cells Background subtracted
% IFNγ+ GZMB+ CD8+ T cells Background subtracted
% IFNγ+ PRFN+ CD8+ T cells Background subtracted
A
B
C
D
C
Stimulated
Unstim
No block
Class I Ab
Class II Ab
Response Magnitude SFC/million PBMCs
>0.9999
0.0142
No block
Class I Ab
Class II Ab

Figure S3

Figure S3: IFNγ-ELISpot assay to examine human CD8^{+}T responses to HLA Class I restricted APOB peptides **(A)** %Granzyme^{+} IFNγ^{+} CD8^{+}T cells (top) and %Perforin^{+} IFNγ^{+} CD8^{+}T cells (bottom) in unstimulated and individual APOB mesopool stimulated PBMCs. Data from eight independent donors. The representative flow cytometry plots were shown in Figure 3C-D. **(B)** Frequencies (mean ± SEM) of IFNγ^{+} CD8^{+}T cells that co-expressed Granzyme (left), TNFα (middle) or Perforin (right) in response to stimulation with APOB mesopools A (white), B (black), C (light grey) and D (dark grey). Background subtracted (responses in stimulated minus unstimulated set) frequencies are shown. (**C**) PBMCs from 3 healthy donors were each expanded with mesopool A-D separately and each expanded set was restimulated with the cognate mesopool in the presence or absence of the blocking antibodies. Representative images of ELISpot wells (left) and quantification (right) showing IFNγ responses to APOB class-I peptides, either alone or in the presence of pan-HLA-I or pan-HLA-II (DP+DQ+DR) blocking antibodies. Background subtracted values are shown. Pairwise comparisons between unstimulated and stimulated sets (A) were done with Wilcoxon matched-pairs signed rank test. Statistical tests (**C**) were done with the Kruskal-Wallis's test and Dunn's multiple comparison adjustment. Numerical p values are listed at the top of each graph. Data is represented as mean ± SEM in (A, B).

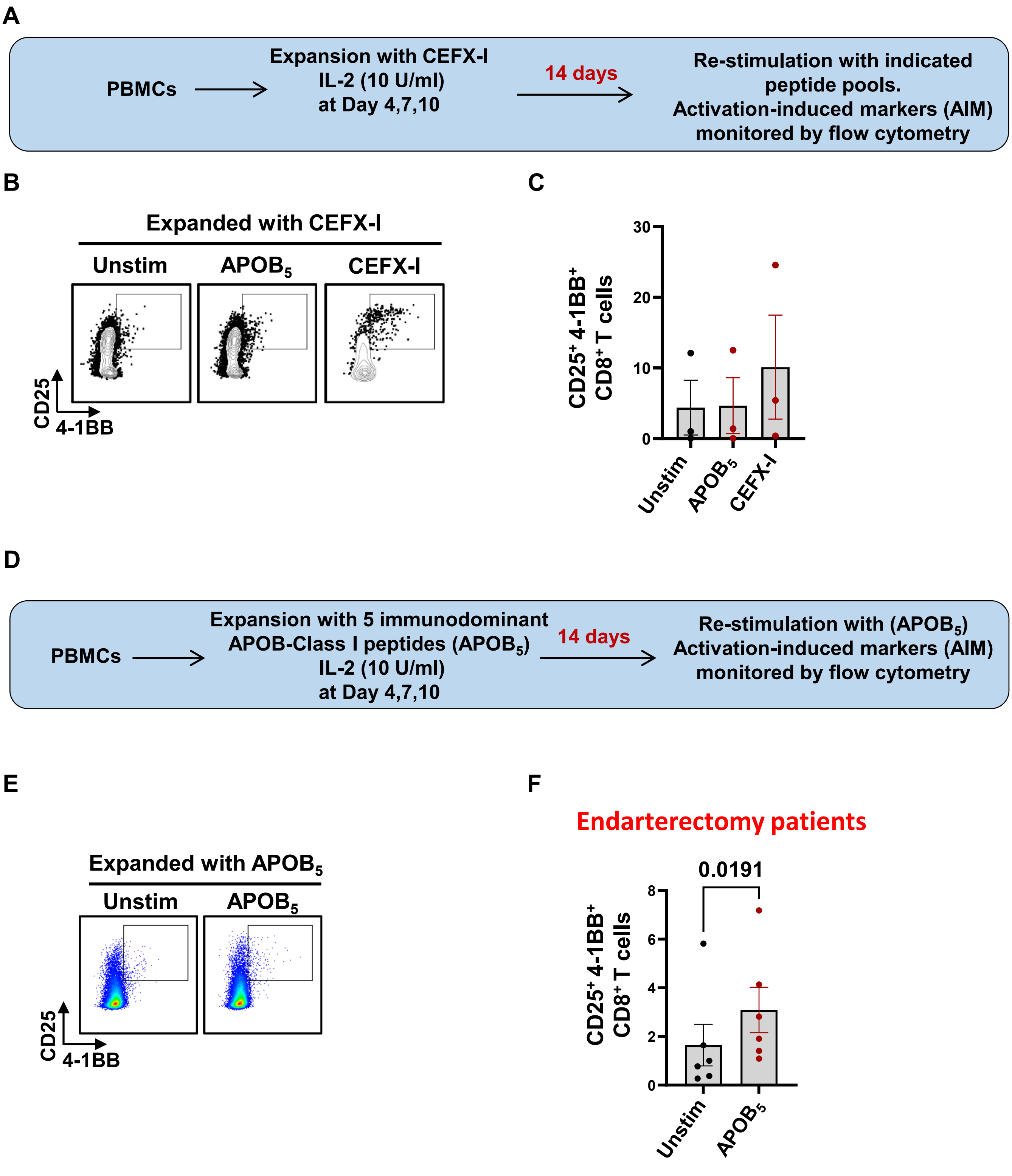

A
PBMCs
Expansion with CEFX-I
IL-2 (10 U/ml)
at Day 4,7,10
14 days
Re-stimulation with indicated peptide pools.
Activation-induced markers (AIM) monitored by flow cytometry
B
Expanded with CEFX-I
Unstim
APOB5
CEFX-I
CD25
4-1BB
C
CD25+ 4-1BB+ CD8+ T cells
30
20
10
0
Unstim
APOB5
CEFX-I
D
PBMCs
Expansion with 5 immunodominant APOB-Class I peptides (APOB5)
IL-2 (10 U/ml)
at Day 4,7,10
14 days
Re-stimulation with (APOB5)
Activation-induced markers (AIM) monitored by flow cytometry
E
Expanded with APOB5
Unstim
APOB5
CD25
4-1BB
F
Endarterectomy patients
0.0191
CD25+ 4-1BB+ CD8+ T cells
8
6
4
2
0
Unstim
APOB5

Figure S4

Figure S4: AIM assay with a positive CEFX-I control and in endarterectomy patients

(A) Schematic diagram depicting the workflow for expansion of human PBMC's with CEFX-I and subsequent restimulation with $APOB_5$ or CEFX-I. Antigen-dependent expression of T cell activation markers was examined using flow cytometry. **(B)** Representative flow cytometry plots showing $CD25^+4\text{-}1BB^+$ $CD8^+$ T cells in unstimulated and stimulated PBMCs (with indicated peptide pools) in healthy subjects. **(C)** Fold change in the frequency of $\%CD25^+4\text{-}1BB^+$ $CD8^+$T cells in stimulated versus unstimulated PBMCs in healthy subjects. Value nearest to the median of % $CD25^+4\text{-}1BB^+$ $CD8^+$T cells in unstimulated control was scaled to 1 and all the other values were plotted as fold change with respect to it. N=3 independent healthy donors . (**D)** Schematic diagram depicting the workflow for expansion and subsequent restimulation of PBMCs from endarterectomy patients with a pool of 5 immunodominant human APOB-derived HLA-A*02:01-restricted peptides ($APOB_5$). Antigen-dependent expression of T cell activation markers was examined using flow cytometry. **(E)** Representative flow cytometry plots showing $CD25^+4\text{-}1BB^+$ $CD8^+$ T cells in unstimulated and $APOB_5$ stimulated PBMCs in patients undergoing endarterectomy**. (F)** Fold change in the frequency of $\%CD25^+4\text{-}1BB^+$ $CD8^+$T cells in $APOB_5$ stimulated versus unstimulated PBMCs in patients undergoing endarterectomy. Value nearest to the median of % $CD25^+4\text{-}1BB^+$ $CD8^+$T cells in unstimulated control was scaled to 1 and all the other values were plotted as fold change with respect to it. N= 6 independent endarterectomy patients. Paired statistical comparisons (F) between unstimulated and stimulated groups were performed using the Wilcoxon matched-pairs signed rank test. Numerical p value is listed at the top of the graph. Data is represented as mean ± SEM.

Major Resources Table

Antibodies

Target antigen	Vendor or Source	Catalog #	Working concentration	Persistent ID / URL
CD14-APC-Cy7	Biolegend	#301819	2µg/ml	https://www.biolegend.com/en-us/products/apc-cyanine7-anti-human-cd14-antibody-3293
CD16-APC-Cy7	Biolegend	#302017	1µg/ml	https://www.biolegend.com/en-us/products/apc-cyanine7-anti-human-cd16-antibody-1904
CD19-APC-Cy7	Biolegend	#302217	0.25µg/ml	https://www.biolegend.com/en-us/products/apc-cyanine7-anti-human-cd19-antibody-1910?GroupID=BLG10095
CD41-APC-Cy7	Biolegend	#303715	0.5µg/ml	https://www.biolegend.com/en-us/search-results/apc-cyanine7-anti-human-cd41-antibody-7113
CD56-APC-Cy7	Biolegend	#318331	0.5µg/ml	https://www.biolegend.com/en-us/search-results/apc-cyanine7-anti-human-cd56-ncam-antibody-7115
CD3-PerCp-Cy5.5	Biolegend	#300429	0.75µg/ml	https://www.biolegend.com/en-us/products/percp-cyanine5-5-anti-human-cd3-antibody-4214
CD4-Pacific Blue	Biolegend	#300524	3.75µg/ml	https://www.biolegend.com/en-us/search-results/pacific-blue-anti-human-cd4-antibody-2850?GroupID=BLG7755
CD8-AF700	Biolegend	#301027	0.75µg/ml	https://www.biolegend.com/en-us/products/alexa-fluor-700-anti-human-cd8a-antibody-3396
CD8-PECy7	Biolegend	#301012	0.75µg/ml	https://www.biolegend.com/en-us/products/pe-cyanine7-anti-human-cd8a-antibody-838
CD40L-PE	Thermo Fisher	#12-1548-42	1µg/ml	https://www.thermofisher.com/antibody/product/CD154-CD40-Ligand-Antibody-clone-24-31-Monoclonal/12-1548-42
CD69-BV650	Biolegend	#310933	5µg/ml	https://www.biolegend.com/en-us/products/brilliant-violet-650-anti-human-cd69-antibody-8345

CD69-AF700	Biolegend	#310922	5µg/ml	https://www.biolegend.com/en-us/products/alexa-fluor-700-anti-human-cd69-antibody-3425
CD25-Pe-Cy7	Biolegend	#302611	1µg/ml	https://www.biolegend.com/en-us/search-results/pe-cyanine7-anti-human-cd25-antibody-1909
CD25-AF647	Biolegend	#302618	0.5µg/ml	https://www.biolegend.com/en-us/products/alexa-fluor-647-anti-human-cd25-antibody-3254
OX-40-APC	Biolegend	#350007	2µg/ml	https://www.biolegend.com/en-us/search-results/apc-anti-human-cd134-ox40-antibody-7090
4-1BB-BV605	Biolegend	#309821	1µg/ml	https://www.biolegend.com/en-us/products/brilliant-violet-605-anti-human-cd137-4-1bb-antibody-12103
CCR7-FITC	Miltenyi	#130-117-812	1µg/ml	https://www.miltenyibiotec.com/US-en/products/cd197-ccr7-antibody-anti-human-reafinity-rea108.html#fitc:30-tests-in-60-ul
CD45RA-AF700	Biolegend	#304119	2.5µg/ml	https://www.biolegend.com/en-us/products/alexa-fluor-700-anti-human-cd45ra-antibody-3421
TNF-BV650	Biolegend	#502937	2µg/ml	https://www.biolegend.com/en-us/products/brilliant-violet-650-anti-human-tnf-alpha-antibody-7680
IFNγ-Pe-Cy7	Biolegend	#502527	1µg/ml	https://www.biolegend.com/en-us/products/pe-cyanine7-anti-human-ifn-gamma-antibody-5939?GroupID=BLG10006
HLA-DR-BV785	Biolegend	#307641	1µg/ml	https://www.biolegend.com/en-us/products/brilliant-violet-785-anti-human-hla-dr-antibody-7975
HLA-DP-PE	BD Biosciences	#566825	1µg/ml	https://www.bdbiosciences.com/en-us/products/reagents/flow-cytometry-reagents/research-reagents/single-color-antibodies-ruo/pe-mouse-anti-human-hla-dp.566825
IFNγ mAb, purified	Mabtech	3420-3-250	5µg/ml	https://www.mabtech.com/products/anti-human-ifn-gamma-antibody-1-d1k-purified-3420-3

IFNγ mAb, biotinylated	Mabtech	3420-6-250	1μg/ml	https://www.mabtech.com/products/anti-human-ifn-gamma-antibody-7-b6-1-biotinylated-3420-6
CD40/TNFRSF5	Novus Biologicals	NBP2-52697	1μg/ml	https://www.novusbio.com/products/cd40-tnfrsf5-antibody-g285_nbp2-52697
purified anti-human HLA-DR, DP, DQ (clone Tu39)	BD Biosciences	555557	20μg/ml	https://www.bdbiosciences.com/en-us/products/reagents/flow-cytometry-reagents/research-reagents/single-color-antibodies-ruo/purified-mouse-anti-human-hla-dr-dp-dq.555557

Other (Peptide pools, cell culture reagents, assay kits, buffers and reagents, software)

Description	Vendor	Catalog #	Persistent ID / URL
CEFX Ultra SuperStim Pool MHC-I Subset	JPT Peptide Technologies	PM-CEFX-4	https://www.jpt.com/cefx-ultra-superstim-pool-mhc-subset/PM-CEFX-4
Phytohemagglutinin-L	eBioscience	00-4977-03	https://www.thermofisher.com/order/catalog/product/00-4977-03
ImmunoCult™ Human CD3/CD28 T Cell Activator	Stemcell	10971	https://www.stemcell.com/products/immunocult-human-cd3-cd28-t-cell-activator.html
Dimethyl sulfoxide	Millipore Sigma	D2650	https://www.sigmaaldrich.com/US/en/product/sigma/d2650
Ficoll® Paque Plus	Millipore Sigma	GE17-1440-03	https://www.sigmaaldrich.com/US/en/product/sigma/ge17144003
CryoStor® CS10	Stemcell	07930	https://www.stemcell.com/cryostor-cs10.html
TexMACS™	Miltenyi Biotec	130-097-196	https://www.miltenyibiotec.com/US-en/products/texmacs-medium.html#gref
Protein Transport Inhibitor	eBioscience	00-4980-03	https://www.thermofisher.com/order/catalog/product/00-4980-03
Ghost Dye™ Violet 510	Tonbo	13-0870-T500	https://tonbobio.com/products/ghost-dye-violet-510
Live/Dead fixable blue stain	Thermofisher	L23105	https://www.thermofisher.com/order/catalog/product/L23105

IC Fixation Buffer	eBioscience	00-8222-49	https://www.thermofisher.com/order/catalog/product/00-8222-49
Permeabilization Buffer (10X)	eBioscience	00-8333-56	https://www.thermofisher.com/order/catalog/product/00-8333-56
UltraComp eBeads™	Invitrogen	01-2222-42	https://www.thermofisher.com/order/catalog/product/01-2222-42
Bovine Serum Albumin	Millipore Sigma	A9647	https://www.sigmaaldrich.com/US/en/product/sigma/a9647
TWEEN® 20	Millipore Sigma	P1379	https://www.sigmaaldrich.com/US/en/product/sial/p1379
N,N-Dimethylformamide	Millipore Sigma	D4551	https://www.sigmaaldrich.com/US/en/product/sigma/d4551
3-Amino-9-ethylcarbazole	Millipore Sigma	A6926	https://www.sigmaaldrich.com/US/en/product/sigma/a6926
VECTASTAIN® Elite ABC-HRP Kit, Peroxidase	Vector laboratories	PK-6100	https://vectorlabs.com/products/abc-kits/vectastain-elite-abc-hrp-kit-standard
Hydrogen peroxide solution	Millipore Sigma	H1009	https://www.sigmaaldrich.com/US/en/product/sigma/h1009
Human IL-2 Recombinant Protein, PeproTech®	Thermofisher	200-02-50UG	https://www.thermofisher.com/proteins/product/Human-IL-2-Recombinant-Protein/200-02-50UG
FlowJo	Becton Dickinson (BD)		
GraphPad Prism version 10	Graphpad		
Immune epitope database	Supported by NIAID		www.iedb.org
